# Asymmetric tetramer metasurface sensor governed by quasi-bound states in the continuum

**DOI:** 10.1515/nanoph-2023-0003

**Published:** 2023-03-06

**Authors:** Yi Zhou, Man Luo, Xuyang Zhao, Yuxiang Li, Qi Wang, Zhiran Liu, Junhong Guo, Zhihe Guo, Junjie Liu, Xiang Wu

**Affiliations:** Key Laboratory of Micro and Nano Photonic Structures (Ministry of Education), Shanghai Engineering Research Centre of Ultra Precision Optical Manufacturing, Department of Optical Science and Engineering, School of Information Science and Technology, Fudan University, Shanghai 200433, P. R. China

**Keywords:** bound states in the continuum, high-Q resonances, optical sensors

## Abstract

Asymmetric metasurfaces supporting quasi-bound states in the continuum (BICs) with high Q-factors and strong light–matter interaction properties are attractive platforms for label-free biosensing applications. Recently, various meta-atom geometries have been exploited to support sharp high-Q quasi-BIC resonance. However, which meta-atom design may be a better practical choice remains unclear. Here, we compared several established meta-atom designs to address this issue by conducting an extensive theoretical discussion on sensing capability and fabrication difficulty. We theoretically revealed that the tetramer meta-atom geometry produces a higher surface sensitivity and exhibits a larger size-to-wavelength ratio than other meta-atom schemes. Furthermore, we found that metasurfaces with a higher depth considerably enhance surface sensitivity. The performance of two asymmetric tetramer metasurfaces (ATMs) with different heights was demonstrated experimentally. Both shallow and thick ATM structures exhibit sharp high Q-factor resonances with polarization-insensitive features. Notably, the surface sensitivity is 1.62 times for thick ATM compared to that for shallow ones. The combination of properties opens new opportunities for developing biosensing or chemical-sensing applications with high performance.

## 1 Introduction

Optical biosensors have gained increasing interest owing to their ability to offer label-free and real-time detection of biomarkers with high sensitivity [1–3]. Among various optical approaches, nanophotonics resonators stand out among others as they exhibit strong light–matter interactions and their ultra-sensitivity for change in refractive index [4, 5]. A popular class of nanophotonics such as photonic crystal (PhC) cavity [6], surface plasmonic resonance (SPR) [7, 8], and guided mode resonance (GMR) [1, 9, 10] have been extensively investigated and exploited in a biosensor. Recently, the performance of these nanophotonic devices has been greatly boosted with the help of novel detection methods [11–14], materials [15, 16], and surface functionalities [17, 18]. These efforts majorly address the limitations of conventional biosensor technologies and make nanophotonic devices a powerful platform for advanced biosensing.

However, these mainstream nanophotonics resonators have some drawbacks. For instance, the PhC cavity can offer high Q-factor resonance (>10^4^); however, they require delicate alignment to couple light into the in-plane waveguide, restricting its potential for point-of-care applications [9, 19]. Conversely, although GMR (or SPR) can be conveniently accessed from free space, they typically produce moderate (or low) Q-factor resonance (<10^3^), restricting their limits of detection, which may hamper detections at a very low concentration [9, 20, 21].

Bound states in the continuum (BICs), a recently emerging nanophotonic resonator [22–30], can address these disadvantages. BICs are special nonradiative electromagnetic states lying in the continuum part of the radiation spectrum, which have infinite lifetimes (Q-factor) in theory [22–24]. Practically, true BICs transform into quasi-BICs with a finite yet high Q-factor owing to the limitations of fabrication, material absorption, and other perturbations [31]. Such quasi-BICs also exhibit extraordinary optical properties for various applications, such as low-threshold laser [32–34], enhanced nonlinear interactions [35–38], and ultrasensitive sensing [13, 14, 39], [40], [41], [42], [43]. Particularly, quasi-BICs can freely couple with free-space light and offer high Q-factor resonance even with small size. Hence, quasi-BICs open new opportunities for developing high-performance, ultracompact, and multiplexed sensing applications.

Recently, researchers have revealed that metasurfaces with broken in-plane symmetry could support symmetry-protected (SP) quasi-BICs as a function of the asymmetric degree (*α*) of constituent meta-atoms [44]. Metasurfaces comprise subwavelength building blocks with artificial arrangements, and their optical properties can be modulated by controlling the shape and geometric parameters of meta-atoms [4]. Owing to this extraordinary flexibility, various meta-atom geometries have been proposed to support the quasi-BICs mode. Examples include monomers (nanodiscs with asymmetric holes [38], crescent shape [40], and T-shaped pillars [35]), dimers (tilted silicon-bar pairs [13, 14], double split-ring structures [27], and asymmetric bar pairs [37]), trimers (asymmetric double-gap split-ring resonators [45]), and tetramers (zigzag elliptical cylinders [46]) of meta-atoms. These different meta-atom geometries have provided attractive experimental results. However, which meta-atom geometry may be a better practical choice remains unclear.

The sensing ability and fabrication difficulties are critical in practical applications. While evaluating the performance of nanophotonic biosensors, the figure of merit is mainly considered [7, 18], which is typically defined as the ratio of bulk sensitivity (*S*_bulk_) to the resonance linewidth (or *S*_bulk_ × *Q* [20]). As for quasi-BICs enabled metasurfaces, the Q-factor is tunable (e.g., 10^3^–10^6^), which is mainly controlled by the asymmetric degree of meta-atoms *α*. Hence, one should pay more attention to sensitivity enhancement, especially surface sensitivity (*S*_surface_), as *S*_surface_ is more applicable in biosensing applications [47]. Meanwhile, symmetry-breaking metasurfaces with the operating wavelength in visible and near-infrared spectral regions are commonly fabricated using the electron-beam lithography (EBL) technique [35, 36, 38, 40, 41, 43, 45]. Hence, a meta-atom with a large defect size is favorable as it exhibits strong tolerance toward fabrication imperfections and reduces production costs. However, keeping a larger defect and relatively large asymmetric degree increases the overall structure size (i.e., period of the unit cell), affording a higher operating wavelength (e.g., 1.55 μm). However, this higher wavelength range may not be suitable for sensing and bioimaging [48]. Hence, a larger defect size of the meta-atom with the same (or even lower) spectral ranges is necessary.

Here, we choose a polarization-insensitive tetramer meta-atom design with a larger size/wavelength ratio. Although such tetramer meta-atoms with polarization-insensitive designs have been investigated recently, most of them are based on theoretical simulation [49–54], and the remainder are tested in the microwave region [55, 56]. Recently, Pravin Vaity et al. studied them in the visible spectral range. The Q-factor was found to be moderate (∼1000), and their biosensing performance remains elusive [57].

Herein, we theoretically and experimentally evaluate a tetramer metasurface with dual-polarization-insensitive resonance governed by quasi-BICs. Compared with other meta-atom designs, asymmetric tetramer metasurfaces (ATMs) exhibit a higher size-to-wavelength ratio (*ζ* = 0.24) (see Table S1, Supplementary Material), which is favorable for practical fabrications. We theoretically explain that this larger size/wavelength ratio property is owing to the excitation of the (1,1) resonant modes, while the primary works are commonly governed by the (1,0) mode. The resonant modes in ATMs also exhibit higher *S*_surface_ than other meta-atom designs owing to their larger surface profile. The performance of two types of ATM structures (shallow and thick ATM) was experimentally evaluated. For shallow ATMs, high Q-factors up to 6061 and 9025 were obtained in the air and liquid environment, respectively. The thick ATM exhibits a higher *S*_bulk_ reaching 171.43 nm/RIU and a higher *S*_surface_ with a more pronounced shift than the shallow ATM. These results suggest that our devices could be useful in highly sensitive biomolecule detection and other potential applications.

## 2 Theoretical analysis

### 2.1 Design and characterization

**Figure 1:**
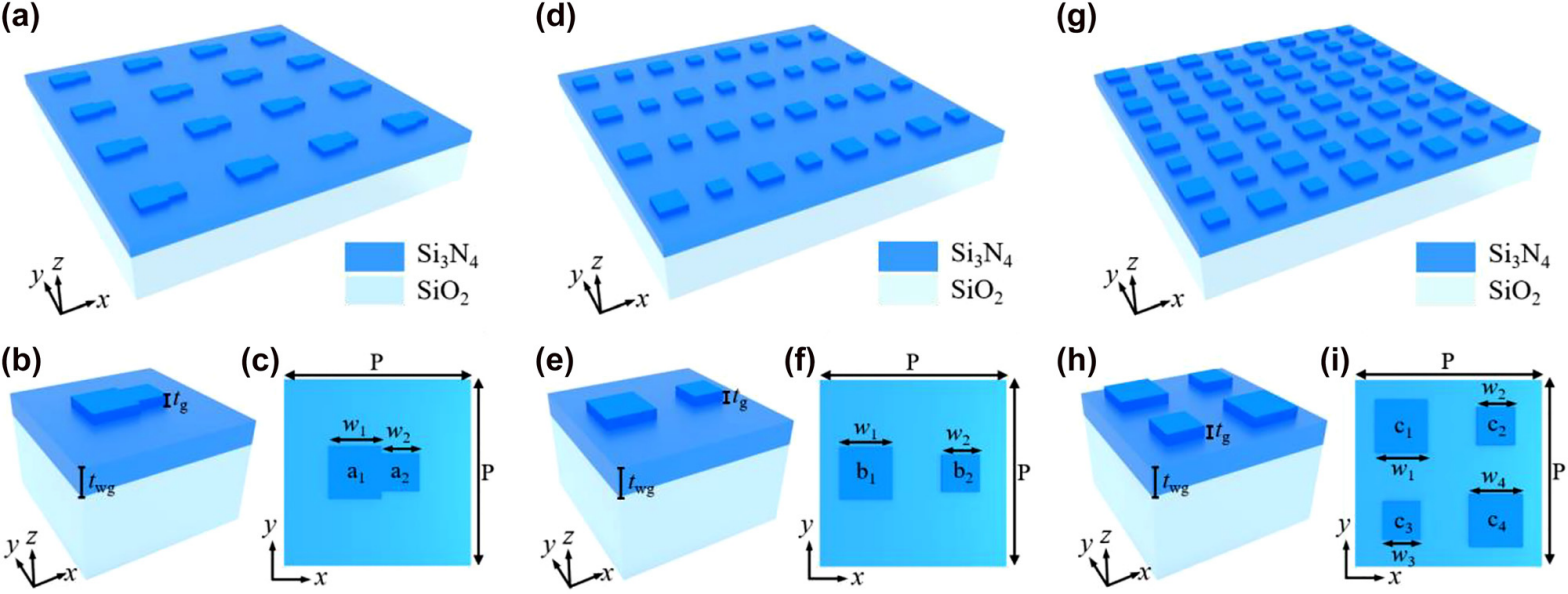
Structure of the asymmetric metasurfaces. (a) Schematic of the Si_3_N_4_ monomer metasurface array on a quartz substrate. (b) Side and (c) top views of a unit cell of the monomer metasurface. (d–f) Schematic of the Si_3_N_4_ dimmer metasurface. (g–i) Schematic of the Si_3_N_4_ tetramer metasurface.

Three different meta-atom (i.e., monomer, dimmer, and tetramer) geometries are used to numerically evaluate their optical properties. The schematic of the designed metasurfaces is shown in Figure 1. Monomer, dimmer, and tetramer meta-atoms are selected for comparison. Cuboids were used as the fundamental building blocks, and the period (*P*) of metasurfaces was fixed at 800 nm. The monomer meta-atom comprised two cuboids (*a*_1_ and *a*_2_) in contact with each other (Figure 1a–c), while the two cuboids (*b*_1_ and *b*_2_) in the dimmer meta-atom had a distance of *P*/2 (Figure 1d–f). The widths of *a*_1_ and *b*_1_ are defined as *w*_1_, and *a*_2_ and *b*_2_ are defined as *w*_2_. Finally, the tetramer meta-atom had four cuboids (*c*_1_, *c*_2_, *c*_3_, and *c*_4_) arranged into a 2 × 2 square supercell, and the center distance between the nearest cuboids is fixed at *P*/2 (Figure 1g–i). The widths of *c*_1_, *c*_2_, *c*_3_, and *c*_4_ are defined as *w*_1_, *w*_2_, *w*_1_, and *w*_2_, respectively.

These metasurfaces are made from silicon nitrogen (Si_3_N_4_) with a thickness of 60 nm (*t*_g_). In contrast with silicon (Si), Si_3_N_4_ gives a high Q-factor resonance in the visible and near-infrared (600–900 nm) region with negligible absorption [11]. However, its moderate refractive index (*n* ∼ 1.9) may lack the structural flexibility to support the desired modes [58]. To overcome this, an unetched Si_3_N_4_ layer with a thickness of 140 nm (*t*_wg_) standing on a quartz substrate as a waveguide layer was used.

### 2.2 Optical properties comparison

**Figure 2:**
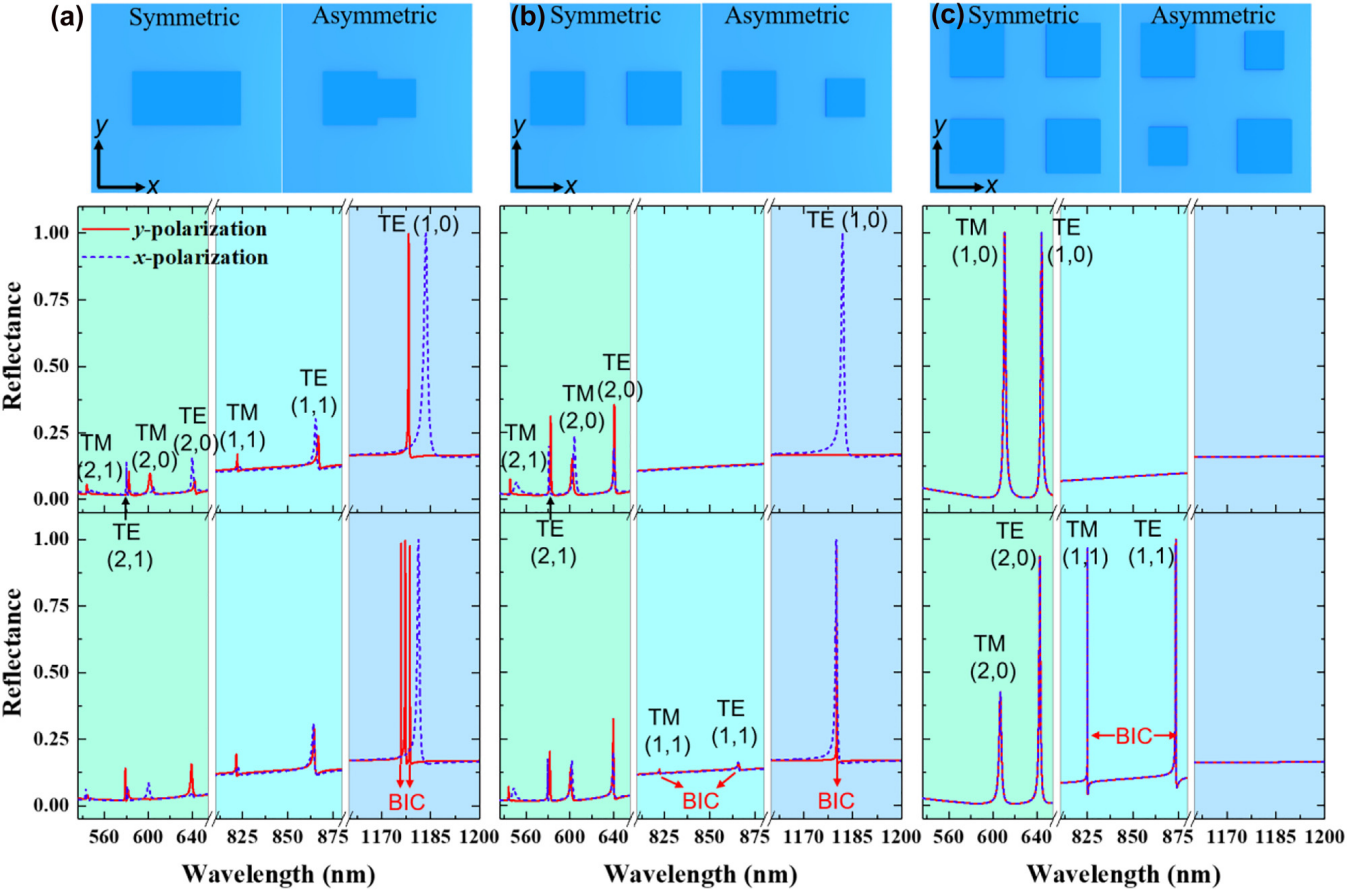
Optical properties of (a) monomer, (b) dimmer, and (c) tetramer unit-atom design in air. Top panel: Schematics of the different meta-atom geometries. Middle panel: Reflection spectra for symmetric metasurfaces under different polarization. Bottom panel: Reflection spectra for asymmetric metasurfaces under different polarization. For symmetric structures, *w*_1_ = *w*_2_ = 280 nm, *t*_g_ = 60 nm, and *t*_*w*g_ = 140 nm. For asymmetric structures, *w*_1_ = 280 nm, *w*_2_ = 200 nm, *t*_g_ = 60 nm, and *t*_*w*g_ = 140 nm.

To understand the optical behavior of these metasurfaces, we utilized the rigorous coupled wave analysis (RCWA) method to calculate the corresponding reflection response. First, we obtained the spectral properties of symmetric and asymmetric metasurfaces for a comprehensive analysis. In the symmetric design, the metasurfaces comprise cuboids with the same width (i.e., *w*_1_ = *w*_2_ = 280 nm), whereas in the asymmetric design, cuboids with different widths are used (i.e., *w*_1_ = 280 nm and *w*_2_ = 200 nm). The polarization angle of 0° (90°) is defined as the *x*-polarized (*y*-polarized) plane wave. Notably, multiple resonant modes can be excited from 535 nm to 1200 nm, primarily arising from the periodic nature of metasurfaces (Figure 2). For a rectangular two-dimensional array under normal incidence, the phase-matching condition is determined by *λ* = *n*_eff_ · *P*/(*i* ^2^ + *j* ^2^)^1/2^, where *n*_eff_ is the effective index of resonance mode and *i* and *j* integers correspond to the orders of diffraction in the *x* and *y* direction, respectively. For simplicity, we only considered a positive number of diffractive orders.

For symmetric monomer metasurfaces, single resonance mode at the window of [1160 nm, 1200 nm] is classified as the TE (1,0) mode; double resonance modes at the window of [810 nm, 880 nm] are classified as TM (1,1) and TE (1,1); and multiple resonance modes at the window of [535 nm, 650 nm] are classified as TM (2,1), TE (2,1), TM (2,0), and TE (2,0) (Figure 2a, middle panel). Notably, these resonance modes are associated with the physical mechanism of the guided mode resonance [59, 60], rather than the scope of the quasi-BICs mode as the structure is symmetric in both *x* and *y* directions. For asymmetric monomer metasurfaces (AMMs), two quasi-BICs can be observed at a window of [1160 nm, 1200 nm] under *y*-polarized light, as the asymmetric structure opens a leaky channel of BIC in the *y* direction (Figure 2a, bottom panel). These quasi-BICs modes excited in the position of (1,0) mode are commonly reported with their defect size/wavelength ratio *ζ* being 0.17. The symmetric dimmer metasurfaces can be considered a grating with a period of *P*/2 and *P* in the *x* and *y* direction, respectively. Hence, the TE (1,0) mode at the window of [1160 nm, 1200 nm] can only be excited under the *x-*polarized light (Figure 2b, middle panel). In asymmetric dimmer metasurfaces (ADMs), although two modes with a similar resonant position at a window of [1160 nm, 1200 nm] can be observed, only the narrow spectrum under *y*-polarized light belongs to the BIC scope (Figure 2b, bottom panel). The polarization must be distinguished practically, which makes device miniaturization and integration inconvenient. Interestingly, two quasi-BICs modes are found at the window of [810 nm, 880 nm], and they also show similar resonant positions (not polarization-insensitive) under differently polarized light. However, their resonant strengths are weak and cannot be used in practical applications. The symmetric tetramer metasurfaces have a grating period of *P*/2 in both the *x* and *y* direction. Hence, the TE (1,0) and TM (1,0) modes are found at the window of [535 nm, 650 nm], and they are also polarization-insensitive (Figure 2c, middle panel). In ATM, all the resonant modes are polarization-insensitive (Figure 2c, bottom panel). Two quasi-BICs modes with strong strength are observed at the window of [810 nm, 880 nm], and the defect size/wavelength ratio of TM (1,1) mode can be up to 0.24, which is 1.42 times than AMM under the same perturbation. Such a large defect size/wavelength ratio is favorable for fabrication.

Moreover, the manipulation of quasi-BICs by fixing the *w*_1_ at 280 nm while varying *w*_2_ from 160 nm to 360 nm (Δ*w* = *w*_1_ − *w*_2_) is demonstrated to further confirm the SP-BIC feature in the designed metasurfaces (see Section S2, Supplementary Material).

### 2.3 Sensing performance comparison

To gauge the sensing performance of these metasurfaces, *S*_bulk_ and *S*_surface_ were calculated. Here, we only considered the quasi-BICs modes (Figure 2). Hence, *y*-polarized light was chosen in calculations. A refractive index of 1.333–1.3624 was considered for the detecting region, and its resonant spectrum was analyzed to calculate *S*_bulk_. For *S*_surface_, a biolayer of 10-nm thickness with a refractive index of 1.5 was assumed to be bound to the surface of the asymmetric metasurface structures (see Figure S5). Here, we defined *S*_bulk_ = Δ*λ*/Δ*n* and *S*_surface_ = Δ*λ*/Δ*l*, where Δ*λ* represents the shift of resonant wavelength owing to the change of the refractive index in the detecting region (Δ*n*) or adsorbate biolayer thickness on the surface of metasurfaces (Δ*l*).

**Figure 3:**
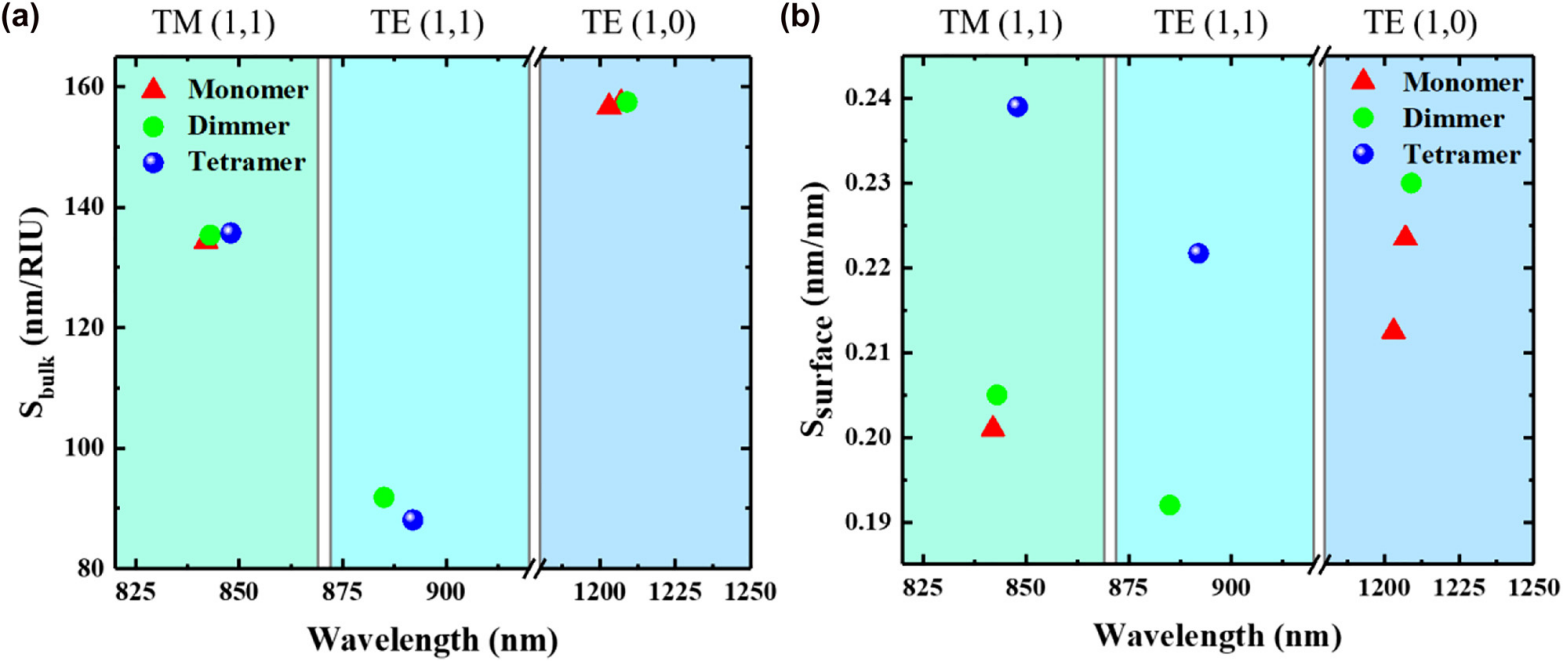
Sensing performance comparison. (a) Calculated *S*_bulk_ and (b) *S*_surface_ for different meta-atom designs. Here, *w*_1_ = 280 nm, *w*_2_ = 200 nm, *t*_g_ = 60 nm, and *t*_wg_ = 140 nm.

Using this method, we obtained a series of comparable *S*_bulk_ values for AMM, ADM, and ATM (Figure 3a). First, a value of ∼135 nm/RIU for TM (1,1) modes was obtained for these three structures. Second, ADM and ATM also showed similar values (91.84 nm/RIU versus 88.09 nm/RIU) under the TE (1,1) modes. Finally, TE (1,0) modes for AMM and ADM give the highest *S*_bulk_ with a value of ∼157 nm/RIU. Hence, the *S*_bulk_ of different metasurfaces is mainly determined by resonant modes rather than structural geometry. Meanwhile, the TM modes have higher sensitivity than TE modes under the same diffraction order, and a higher working wavelength gives higher sensitivity under TE modes.

Further, *S*_surface_ should be considered in biosensing applications. We also compared the *S*_surface_ of different meta-atom designs under the same resonance modes (Figure 3b). It can be observed that ATM has a higher *S*_surface_ than other types of metasurfaces under the TM (1,1) and TE (1,1) modes, which is different from the results of *S*_bulk_. Compared with AMM, an almost 20% improvement (0.239 nm/nm versus 0.201 nm/nm) was achieved by ATM under the TM (1,1) mode. Further, the *S*_surface_ of ATM under the TE (1,1) mode showed a ∼16% enhancement compared with ADM. We speculate that more cuboids in the structure surface bring more side profiles, affording a larger surface area to absorb proteins. This speculation could be easily verified by increasing the height of the metasurfaces, i.e., *t*_g,_ herein (see Section S3, Supplementary Material). It was observed that *S*_surface_ could be improved by increasing *t*_g_ for all meta-atom designs (Figure S6). Notably, the *S*_surface_ of ATM shows a higher increment than others after increasing *t*_g_. In our final optimization, the *S*_surface_ of the TM (1,1) mode for ATM could be up to 0.4 nm/nm (see Section S3, Supplementary Material), which was ∼1.48 times higher than that of AMM and ADM.

Briefly, the *S*_bulk_ is mainly determined by the diffraction order and polarization property rather than the geometry of the meta-atom. Conversely, the meta-atom design will influence *S*_surface_, although the diffraction order and polarization property are fixed.

### 2.4 Electric-field distribution

After theoretically comparing the optical properties of different meta-atom designs, the tetramer metasurface revealed many unique properties than others, such as larger defect size/wavelength ratio *ζ*, higher polarization insensitivity, and higher *S*_surface_. For simplicity, we mainly considered the tetramer metasurface in the following discussions.

**Figure 4:**
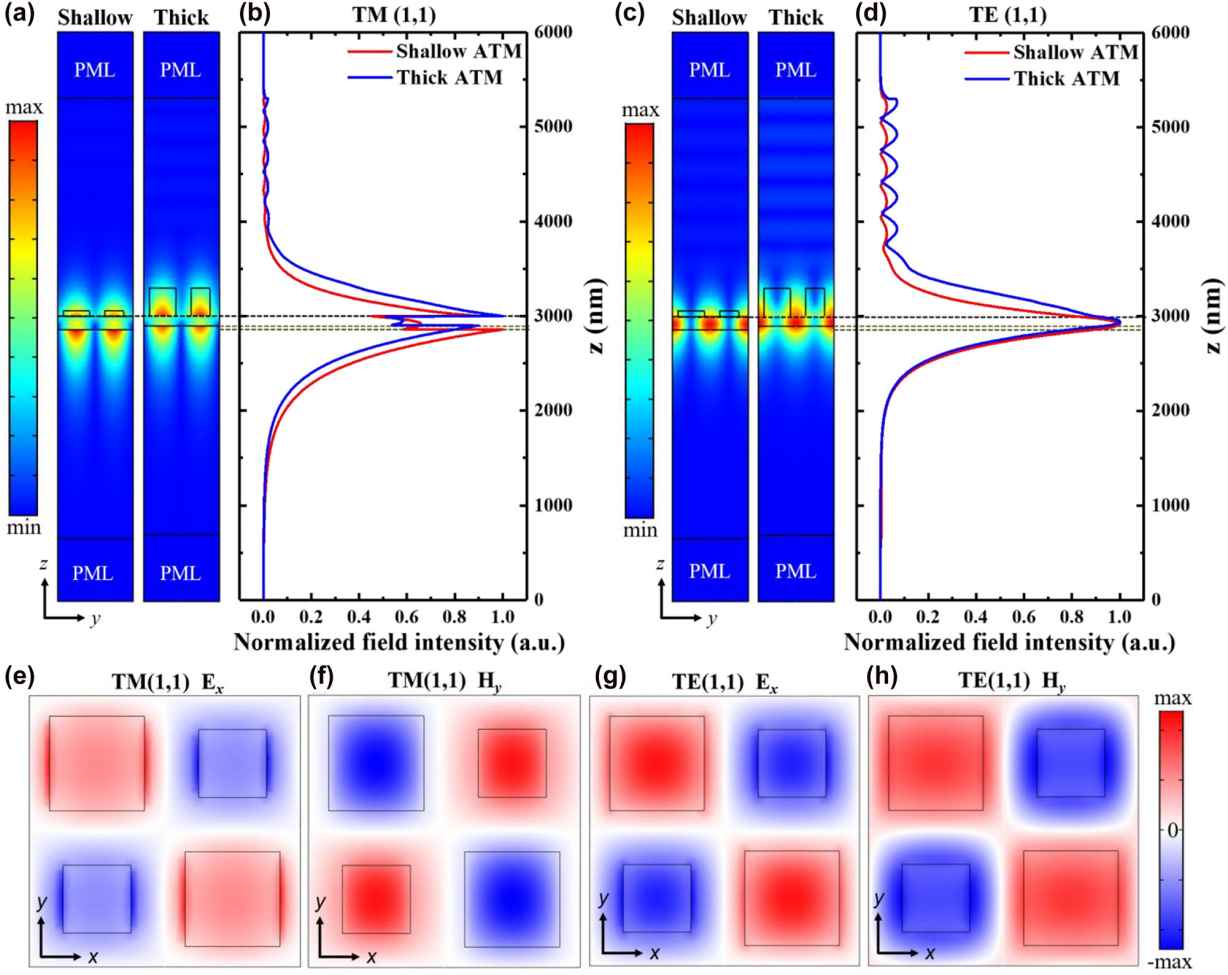
Optical field distribution of ATM structures. (a) Resonance electric-field distribution and (b) normalized field intensity across the *z* axis of ATM structures under TM (1,1) mode. (c) Resonance electric-field distribution and (d) normalized field intensity across the *z* axis of ATM structures under TE (1,1) mode. (e–h) Simulated in-plane field distribution of the resonance mode for ATM structure. For shallow ATM, *w*_1_ = 280 nm, *w*_2_ = 200 nm, *t*_g_ = 60 nm, and *t*_*w*g_ = 140 nm. For thick ATM, *w*_1_ = 280 nm, *w*_2_ = 200 nm, *t*_g_ = 300 nm, and *t*_*w*g_ = 100 nm.

To attain a deeper understanding of the tetramer metasurface, the electric-field distributions are analyzed based on finite-element method. According to the previous analysis, we chose two types of ATM structures, one with a shallow *t*_g_ ∼ 60 nm (called shallow ATM) and the other with a thick *t*_g_ ∼ 300 nm (called thick ATM). For TM (1,1) modes, the electric field is more extended in the structural surface and substrate regions (Figure 4a), which is suitable for sensing. Conversely, the electric field is mainly confined in the waveguide layer for the TE (1,1) mode (Figure 4c), which is favorable for imaging [48]. Figure 4b and d show the electric-field distributions across the *z* axis. It can be observed that the thick ATM brings the optical field toward the sensing region and thus enhances *S*_bulk_. The *E*_*x*_ and *H*_*y*_ components of the two modes show similar antisymmetric distributions along the *x* and *y* axis (Figure 4e–h). If the structure is symmetric, these in-plane fields will be completely out-of-phase and thus well localized in the structure.

## 3 Experimental results

### 3.1 Optical properties of ATM structures in air

**Figure 5:**
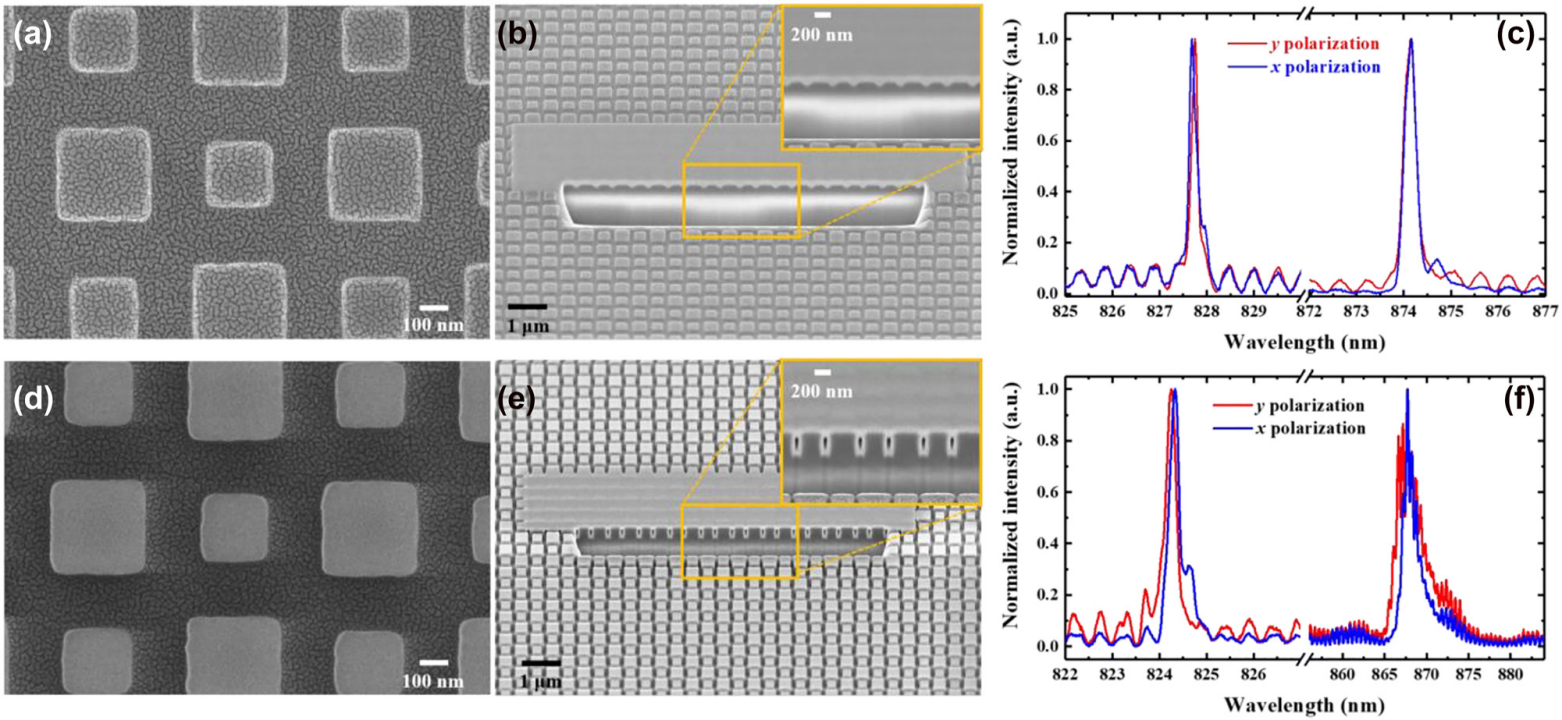
Images and optical properties of ATM structures. (a, d) Top-view SEM images and (b, e) side-view focused ion beam (FIB) cuts of shallow and thick ATM. Inset: FIB cuts in a magnified view. (c, f) Experimental reflection spectrum for shallow and thick ATM in air. The geometric parameters were set as follows: *t*_g_ = ∼60 nm and *t*_*w*g_ = ∼140 nm for shallow ATM; *t*_g_ = ∼300 nm, and *t*_*w*g_ = ∼100 nm for thick ATM. The array size is 500 μm × 500 μm.

We used the EBL to fabricate these structures (see Section S1, Supplementary Material). Scanning electron microscope (SEM) images of the samples are shown in Figure 5a and d. Focused ion beam (FIB) cut reveals metasurfaces with different *t*_g_ (Figure 5b and e).

The biomarkers detections were generally demonstrated in a buffer environment. Further, various works measured the targeted biomolecular in a dry state as a larger refractive index contrast brings more sensitivity to a local index change [40]. Hence, we first measured the reflectance spectra of the two ATM structures at normal incidence in air. A polarizer was used to change the polarization direction during measurement. Lorentz fitting was used to obtain the corresponding central wavelengths and linewidth (Q-factors). For shallow ATM structures, the measured wavelengths for TM (1,1) (or TE (1,1)) were 827.7 nm and 827.75 nm (or 874.14 nm and 874.13 nm) under *x* and *y* polarization, respectively, which confirmed that the quasi-BICs mode is polarization-insensitive (Figure 5c). The Q-factor was 6061 and 3495 for TM (1,1) and TE (1,1), respectively. A high Q-factor and dual-polarization insensitivity are required for various applications. The optical property of the thick ATM structure was also characterized under different polarization conditions (Figure 5f). The resonant wavelengths are located at 824.32 nm (867.89 nm) and 824.24 nm (867.99 nm) for TM (1,1) (TE (1,1)) under *x* and *y* polarization, respectively. In this case, the Q-factors were 3588 and 478 for TM (1,1) and TE (1,1), respectively. For comparison, in simulation, the shallow ATM shows a Q-factor of 14,893 and 3802 for TM (1,1) and TE (1,1), respectively (Figure S8a). For thick ATM structure, the simulated Q-factor is 3192 for TM (1,1) mode and 580 for TE (1,1) mode (Figure S8b). These theoretical results match well with the experimental demonstrations.

Additionally, we characterized different fabricated samples and summarized their Q-factors and the wavelength deviation (Figure S10). The average Q-factor for shallow ATM structures was 4624 and 3003 for TM (1,1) and TE (1,1) resonant modes, respectively. For thick ATM structures, the average Q-factor was 2288 for TM (1,1) and 503 for TE (1,1), which was lower than shallow ATM structures. The lower Q-factor can be attributed to a thick ATM structure that brings more confined energy out of the waveguide (Figure 4).

**Figure 6:**
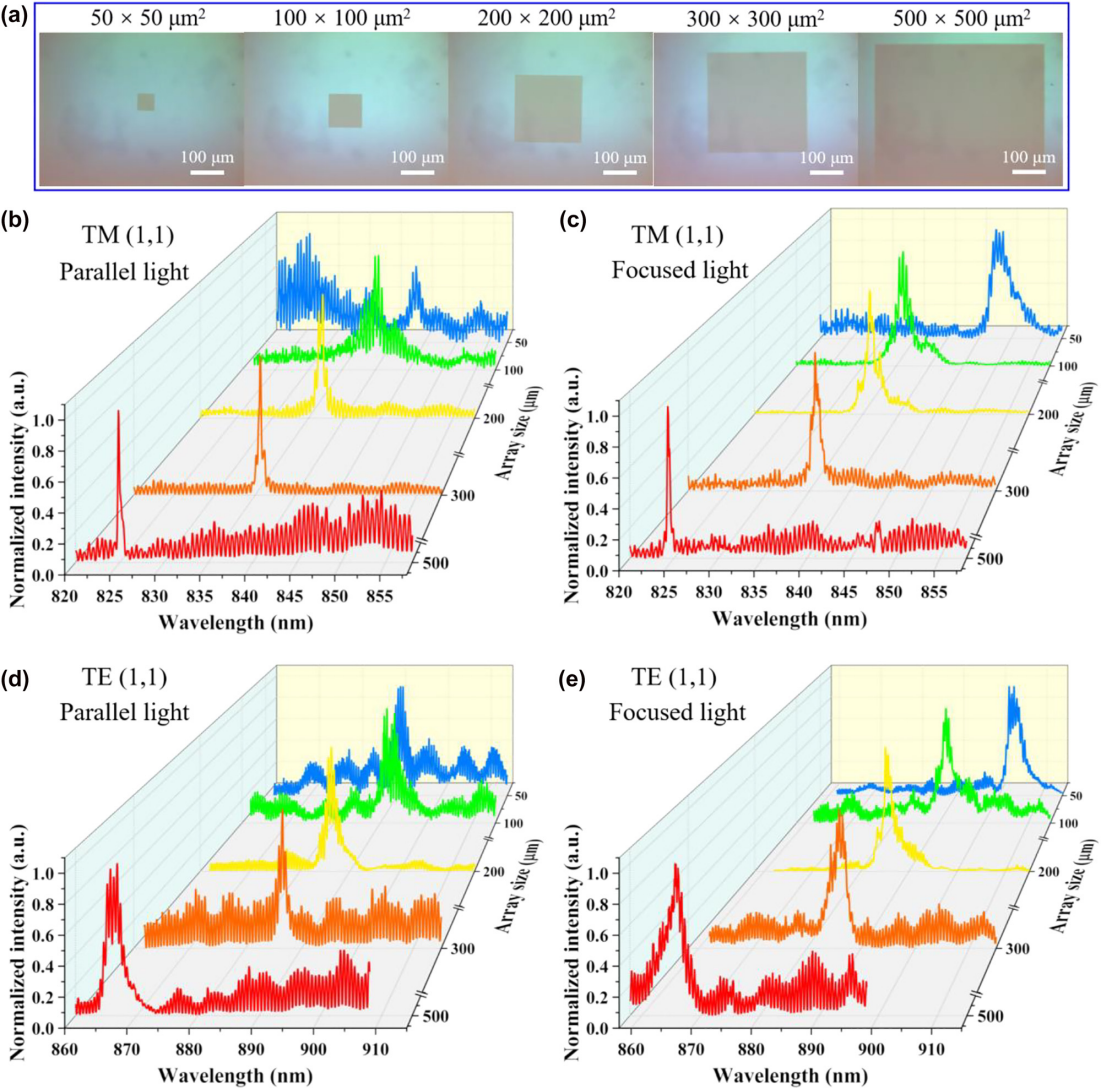
Effects of array size. (a) Microscope images of the thick ATM with different array sizes. The measured spectra of thick ATMs with different array sizes for TM (1,1) mode under (b) parallel and (c) focused incident light. The measured spectra of thick ATMs with different array sizes for TE (1,1) mode under (d) parallel and (e) focused incident light.

Previous works have demonstrated that a larger array size is preferred for obtaining higher *Q* resonance. Here, we also fabricated a series of thick ATM structures with different array sizes (50 μm × 50 μm, 100 μm × 100 μm, 200 μm × 200 μm, 300 μm × 300 μm, and 500 μm × 500 μm) (Figure 6a). Parallel light or focused light was used to illuminate different samples. Figure 6b–e shows the measured spectra under nonpolarized light. For TM (1,1) mode, it can be observed that resonant peaks become narrow as the array size increases (Figure 6b and c). Notably, the extracted Q-factors for the parallel incident light are larger than those for the focused one with the same sample (Figure S11). We note, however, that focused light can produce a smaller spot size combined with stronger spatial light intensity than parallel light, resulting in the reflected signal primarily coming from the metasurface region rather than the background noise. Hence, focused light can produce a stronger resonance signal even with a small sample (50 μm × 50 μm). Meanwhile, the Q-factors of TE (1,1) mode appeared to be independent of the array size (Figure 6d and e and Figure S11). The reason can be attributed to their relatively low Q-factors in theory (*Q*_theory_ = 580), where the overall experimental Q-factor (defined as *Q*_total_^−1^ = *Q*_theory_^−1^ + *Q*_error_^−1^) is mainly determined by the theoretical design (*Q*_theory_) rather than imperfect fabrications (*Q*_error_, including roughness, nonuniformity, array size, and so on) [61]. Therefore, simply increasing the array size may not be sufficient to significantly improve the realistic Q-factors of TE (1,1) mode. In addition, the recorded spectra exhibit an obvious interference signal, especially in a low *Q* resonant peak, owing to the coherent nature of the supercontinuum source.

### 3.2 Refractive index sensing

**Figure 7:**
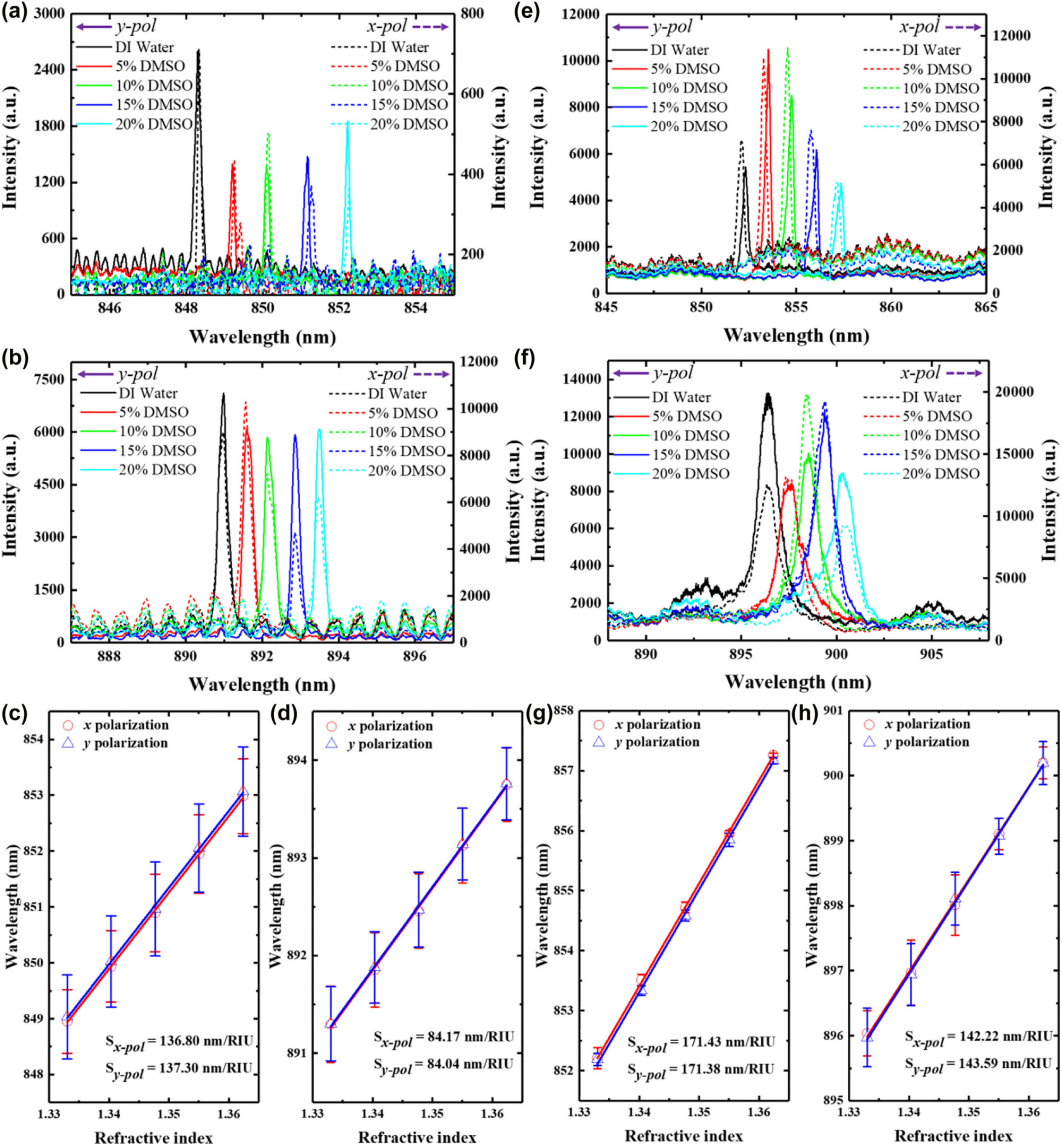
Reflectance spectra of TM (1,1) and TE (1,1) mode for (a, b) shallow ATM and (e, f) thick ATM immersed in DMSO–water mixture with different refractive indices. Measured resonance peaks of TM (1,1) and TE (1,1) mode position as a function of the refractive index of the solutions for (c, d) shallow ATM and (g, h) thick ATM structure.

*S*_bulk_ for both shallow and thick ATM structures was characterized by assembling a microfluidic channel on the top of the structures. We measured the reflectance spectra of resonant quasi-BICs modes associated with different concentrations of dimethyl sulfoxide (DMSO) and deionized water mixtures (0%, 5%, 10%, 15%, and 20%), as shown in Figure 7. An obvious red shift with the increment of the concentrations of DMSO was observed. Particularly, for a given refractive index, these peaks show a negligible difference in the resonant wavelengths under different polarization, which confirms the polarization-insensitivity of ATM structures in the solution. The extracted wavelength positions of the two quasi-BICs under *x* and *y* polarization are plotted against the refractive index of detecting solutions in Figure 7c, d, g, and h for shallow ATM and thick ATM, respectively. Here, we obtained an *S*_bulk_ of 136.80 nm/RIU (137.30 nm/RIU) for shallow ATM under TM (1,1) mode and a high Q-factor of 9025 (5335) under *x* (*y*) polarization. Meanwhile, the TE (1,1) mode of shallow ATM attains an *S*_bulk_ of 84.17 nm/RIU (84.04 nm/RIU) and a high Q-factor of 3828 (3757) under *x* (*y*) polarization. However, the thick ATM shows a higher *S*_bulk_ of ∼171 nm/RIU and ∼142 nm/RIU for TM (1,1) and TE (1,1) modes, respectively (Figure 7g and h). From an evanescent field point of view, the thick ATM structure has a larger evanescent field distribution in the sensing region than shallow ones (Figure 4b and e). Further, the shallow ATM structure exhibits larger evanescent energy in the waveguide layer than the thick structure. In TM (1,1) (or TE (1,1)) mode, ∼10.6% (or ∼38.7%) of evanescent energy is stored in the waveguide layer for shallow ATM structure, only 5.5% (or ∼24%) for thick ATM structure, indicating a higher Q-factor for shallow structure. Here, the evanescent field region is defined as the region where the normalized intensity drops to a value of 1/*e*.

### 3.3 Biosensing

To characterize the proposed ATM structures as biosensors and demonstrate biosensing performance, we chose a high-affinity biotin–streptavidin (SA) bioassay [40, 43]. Furthermore, shallow and thick ATM structures were used to compare the experimental *S*_surface_. Before biodetections, the metasurface chips were treated with piranha solution and oxygen plasma to generate hydroxyl groups (–OH) on the Si_3_N_4_ surface (see Section S1, Supplementary Material). Next, the metasurfaces chips were immersed in an ethanol solution of (3-aminopropyl) triethoxysilane (APTES, 2% v/v) for 2 h to treat the chip surface with amino groups (–NH_2_). Afterward, the chips were assembled on a microfluidic channel for biosensing measurements (see Section S1, Supplementary Material).

**Figure 8:**
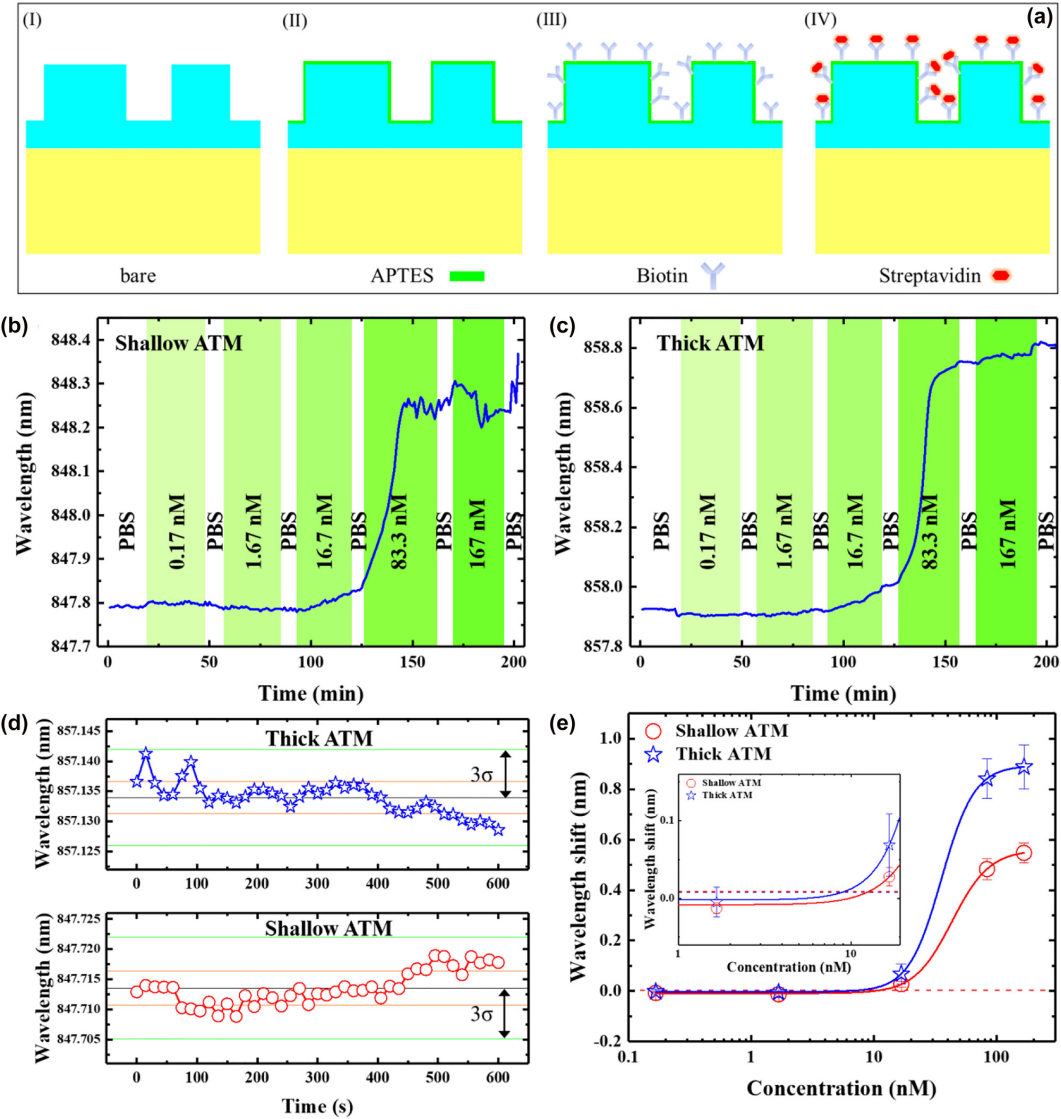
Specific binding of streptavidin to the ATM structures. (a) Schematic of the surface functionalization process. Real-time biomolecular interaction response of TM (1,1) mode for (b) shallow ATM and (c) thick ATM structure. (d) Resonant wavelength versus time in PBS buffer. The orange and green lines indicate one and three standard deviations (*σ*) away from the mean, respectively. (e) Streptavidin calibration curves for shallow ATM (red) and thick ATM (blue). The red and blue horizontal dashed lines mark the detection limit for shallow ATM and thick ATM, respectively.

The experiments were performed after incubating the channels in a sulfo–NHS–biotin solution for 2 h. Subsequently, the sensor chip was washed with phosphate-buffered saline (PBS) buffer for at least 30 min. Different concentrations of SA solution were pumped into the chip to conjugate SA with biotin. PBS was used to remove the unbound SA protein after each incubation. It is worth noting that the TM modes exhibit more advantageous sensing properties than TE modes, such as higher surface sensitivity, higher Q-factors, and more extended field distributions. Hence, for both shallow and thick ATM structures, the resonant peaks of TM (1,1) were recorded in real time to dynamically monitor the specific binding between Biotin and SA (Figure 8b and c). The detection limit for both ATM structures was determined as the minimum concentration that would produce a wavelength shift corresponding to three times the standard deviation (3*σ*) [10]. As shown in Figure 8d, the 3*σ* of resonant mode was measured to be 8.46 and 7.95 pm for shallow and thick ATM structures, respectively. The average response shifts at each concentration were extracted and were fitted with a four-parameter logistic equation [10, 40], as shown in Figure 8e. According to the calibration curve and 3*σ*, we found a detection limit of 12.6 nM and 9 nM for shallow and thick ATM structures, respectively. Although such a result is lower than previous reported sensors based on quasi-BICs, these reports were demonstrated in a dry state rather than the solution environment [40, 43]. In the future, novel surface functionalities and self-referencing technologies can be used to enhance the biosensing performance of ATM structures [12, 17, 48].

Particularly, thick ATM structures showed higher resonance shifts than shallow ones for the same SA protein concentration (Figure 8e). Theoretically, *S*_surface_ of the thick ATM structure was 1.67 times higher than that of shallow ATM (0.4 nm/nm versus 0.239 nm/nm, Figure 3b and Figure S7a). The experimental *S*_surface_ values of the thick ATM were 2.48, 1.74, and 1.62 times higher than those of the shallow ATM at concentrations of 16.7 nM, 83.3 nM, and 167 nM, respectively. This result is consistent with the theoretical analysis, particularly in biodetections at high concentrations.

## 4 Conclusions

We investigated the optical performance of different metasurfaces based on mainstream meta-atom geometries. We found that ATM can excite strong quasi-BIC resonance with polarization insensitivity, which is associated with the (1,1) resonant mode. Compared with other meta-atom designs, such (1,1) resonant mode enabled the metasurface to exhibit a higher size-to-wavelength ratio (*ζ* = 0.24), which is favorable for practical fabrication. It is further demonstrated that ATM structures exhibit higher *S*_surface_ than other meta-atom designs, especially for structures with higher depth. Shallow ATM structures obtained a sensitivity of 136.80 nm/RIU and a high Q-factor of up to 6061 and 9025 in the air and liquid environment, respectively. Thick ATMs exhibit a higher *S*_bulk_ reaching 171.43 nm/RIU and a higher *S*_surface_ with a more pronounced shift compared to the shallow ATM. In the future, novel surface functionalities and self-referencing technologies can be used to enhance the biosensing performance of ATM structures.

## Supplementary Material

Supplementary Material Details
