# Supplementary material for: Asymmetric tetramer metasurface sensor governed by quasi-bound states in the continuum
Source: Nanophotonics. 2023 Mar 6;12(7):1295–307. doi: 10.1515/nanoph-2023-0003 (PMC11636488; doi:10.1515/nanoph-2023-0003)
Supplement: Supplementary file 1 — Supplementary Material Details [file j_nanoph-2023-0003_suppl_001.docx]

Supplementary Material

**Asymmetric tetramer metasurface sensor governed by quasi bound states in the continuum**

*Yi Zhou**^1^, Man Luo^1^, Xuyang Zhao^1^, Yuxiang Li^1^, Qi Wang^1^, Zhiran Liu^1^, Junhong Guo^1^, Zhihe Guo^1^, Junjie Liu^1^, and Xiang Wu^1,^**

^1^Key Laboratory of Micro and Nano Photonic Structures (Ministry of Education), Shanghai Engineering Research Centre of Ultra Precision Optical Manufacturing, Department of Optical Science and Engineering, School of Information Science and Technology, Fudan University, Shanghai 200433, P. R. China.

E-mail: [wuxiang@fudan.edu.cn](mailto:wuxiang@fudan.edu.cn)

**Section S1. Methods**

***Simulations***: Rigorous coupled wave analysis (RCWA) was used to calculate the reflection response (Rsoft, DiffractMOD). The finite-element method based on a commercial software (COMSOL Multiphysics) was used to calculate the corresponding electromagnetic field. Floquet periodic boundary conditions were applied in the *x* and *y* directions, and perfectly matching layers were employed in the *z* direction. In all the simulations, the refractive index of Si_3_N_4_ and quartz was set as 1.9 and 1.45, respectively.

***Measurements****:* A home-built setup was used to measure the reflected spectra at normal incidence. A supercontinuum source (470–2400 nm, SC-5-FC, YSL Photonics) was used to emit light via a single-mode optical fiber. The fiber was collimated using a fiber collimator to produce parallel light. Then, the parallel light was passed through a diaphragm with a diameter of 500 μm. The light was normally incident on the ATM metasurfaces. For focused light, a lens with *f* = 40 mm was employed before striking the light on the ATM metasurfaces. Such metasurfaces can reflect light through a 50:50 beam splitter, and then the light is polarized using a Glan polarizer. Finally, the reflected light was collected on the fiber tip connected to a spectrometer (SpectraPro-2750, Acton Research).

***Fabrications***: All the samples were fabricated on 1 cm × 1 cm quartz wafers using EBL. The quartz wafers were cleaned with acetone at 70°C and sonicated for 5 min. Si_3_N_4_ (200/400) nm was deposited on quartz wafers using plasma chemical vapor deposition (PlasmaPro 100 PECVD, OXFORD). Next, an electron beam resist (ZEP520) was spin-coated on the sample at 4000 rpm to obtain a thickness of 360 nm, and the sample was baked at 180°C. EBL was performed on the sample to define the pattern of the material, and then the electron beam resist was developed in the ZDN50 solution. Afterward, the pattern was transferred into the Si_3_N_4_ layer using plasma etching processes with sulfur hexafluoride (SF_6_) and trifluoromethane (CHF_3_) as etchants. The sample was immersed in butanone to remove the residual material.

***Biosensing Preparation***: The sensor chip was cleaned via ultrasonication in ethanol and DI water for at least 10 min and dried with compressed air. Next, the sensor chip was immersed in the piranha solution (H_2_SO_4_: H_2_O_2_ = 3: 1) for 1 h and washed multiple times in DI water to dissipate any organic contamination on the surface and dried. Afterward, oxygen plasma cleaning was used to generate hydroxyl radicals (–OH) on the sensor chip. Immediately, it was immersed in ethanol mixed with (3-aminopropyl)triethoxysilane (APTES, 2% v/v) (Sigma–Aldrich) for 2 h to functionalize the chip surface with amino groups (–NH_2_). Then, it was cleaned with ethanol and dried with compressed air. The sulfo–NHS–biotin (ThermoFisher Scientific) molecules can be captured by NH_2_ groups. The sensor surface was incubated with the sulfo–NHS–biotin solution (2 mg/mL) in PBS buffer in a PDMS microfluidic channel and maintained for 2 h. Then, the biotin-functionalized sensor was washed using phosphate-buffered saline (1 × PBS) buffer (Sigma–Aldrich). Finally, the biotin-functionalized sensor was incubated in different SA (ThermoFisher Scientific) concentrations. A washing process (1 × PBS buffer, Sigma–Aldrich) was performed to remove the unbound protein after each incubation. For both shallow and thick ATM sensors, the experiments were performed independently at least three times.

***Microfluidics channel***: The microfluidic channel was prepared using a molded polydimethylsiloxane (PDMS) elastomer. The PDMS was mixed with a hardener in a ratio of 10:1. The ATM metasurface was fabricated on a quartz substrate 10-mm wide, 10-mm long, and 0.5-mm high. To avoid large gradient changes in the liquid flow, a PDMS hole mold was fabricated as the bottom substrate to place the sensor chip. The hole was 11-mm wide, 11-mm long, and 0.55-mm high. Meanwhile, a 30-mm-long, 0.5-mm-wide, and 0.3-mm-high fluidic channel was designed as a top layer. The inlet and outlet holes were punched through the top PDMS, and Tygon tubing was inserted into the holes. Briefly, the sensor chip was sandwiched between the top fluidic channel and the bottom substrate (Figure S1). Finally, uncured PDMS was used as glue to fix the microfluidic channel. The material was cured at 60°C for at least 2.5 h.


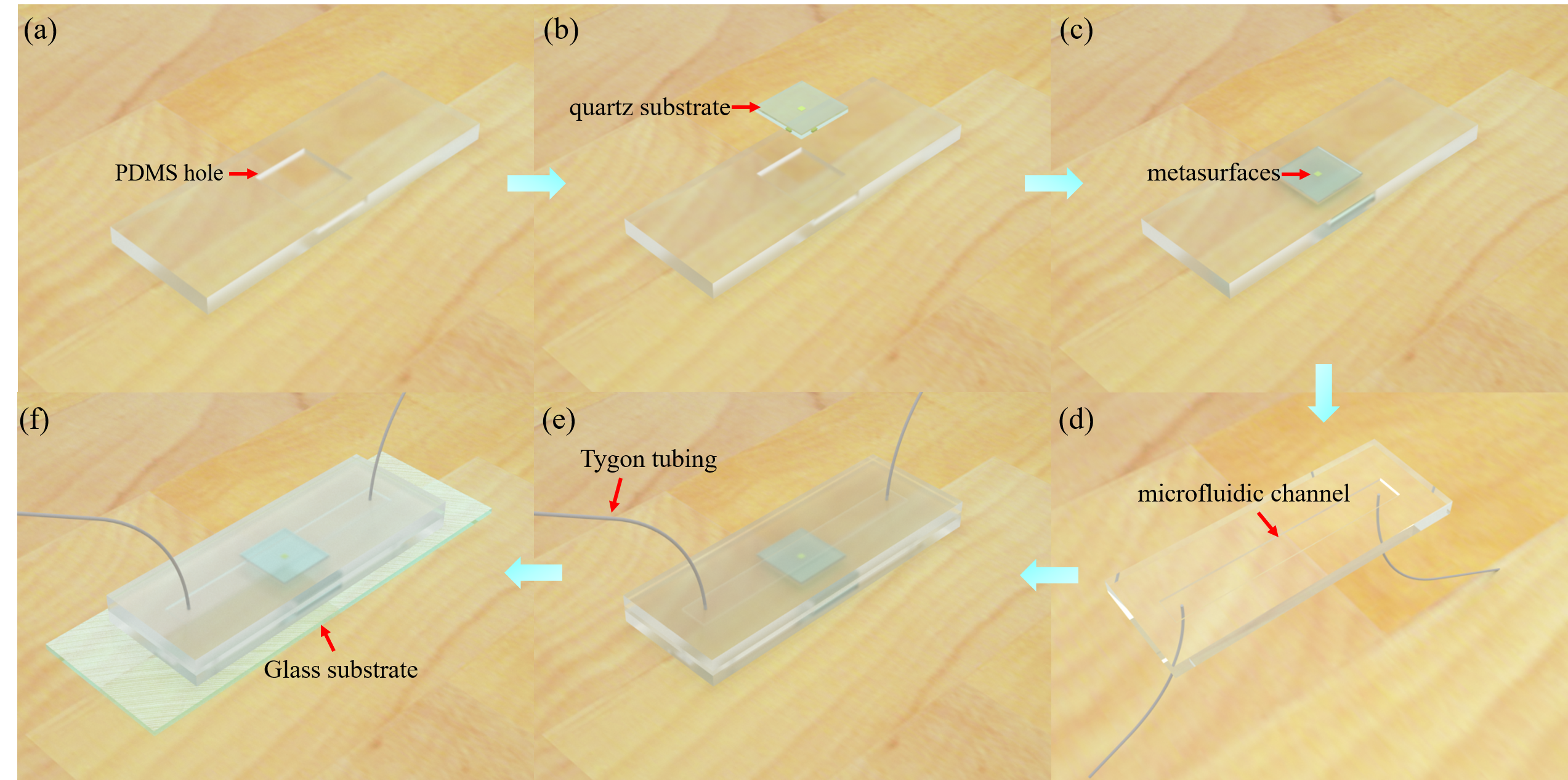


Figure S1. The integration process of metasurfaces chip and PDMS microfluidic channel. (a) A bottom PDMS with a square hole (11 mm wide, 11 mm long, and 0.55 mm high) was fabricated. (b,c) A quartz substrate (10 mm wide, 10 mm long, and 0.5 mm high) with ATM metasurfaces (500 μm × 500 μm) was placed on the bottom PDMS hole. (d) A top PDMS with a microfluidic channel (30 mm long, 0.5 mm wide, and 0.3 mm high) was fabricated. (e) Afterward, the top PDMS was assembled with the bottom PDMS. (f) Finally, the whole PDMS microfluidic channel was placed on a glass substrate.

**Section S2. Manipulation of quasi-BICs for different metasurfaces.**

Figures S2a, S3a, and S4a show the reflection spectra of various metasurfaces under *y*-polarized normal incidence. Narrow reflection peaks become visible in simulations at Δ*w* ≠ 0 nm, which means a transition from an SP-BIC to a quasi-BIC, due to asymmetry. Figures S2b, S3b, and S4b manifest the Q-factors of the quasi-BICs modes as a function of Δ*w*. One can observe that the Q-factors of the quasi-BICs tend to “infinity” at Δ*w* = 0, which is the evidence of SP-BIC feature in the designed metasurfaces.


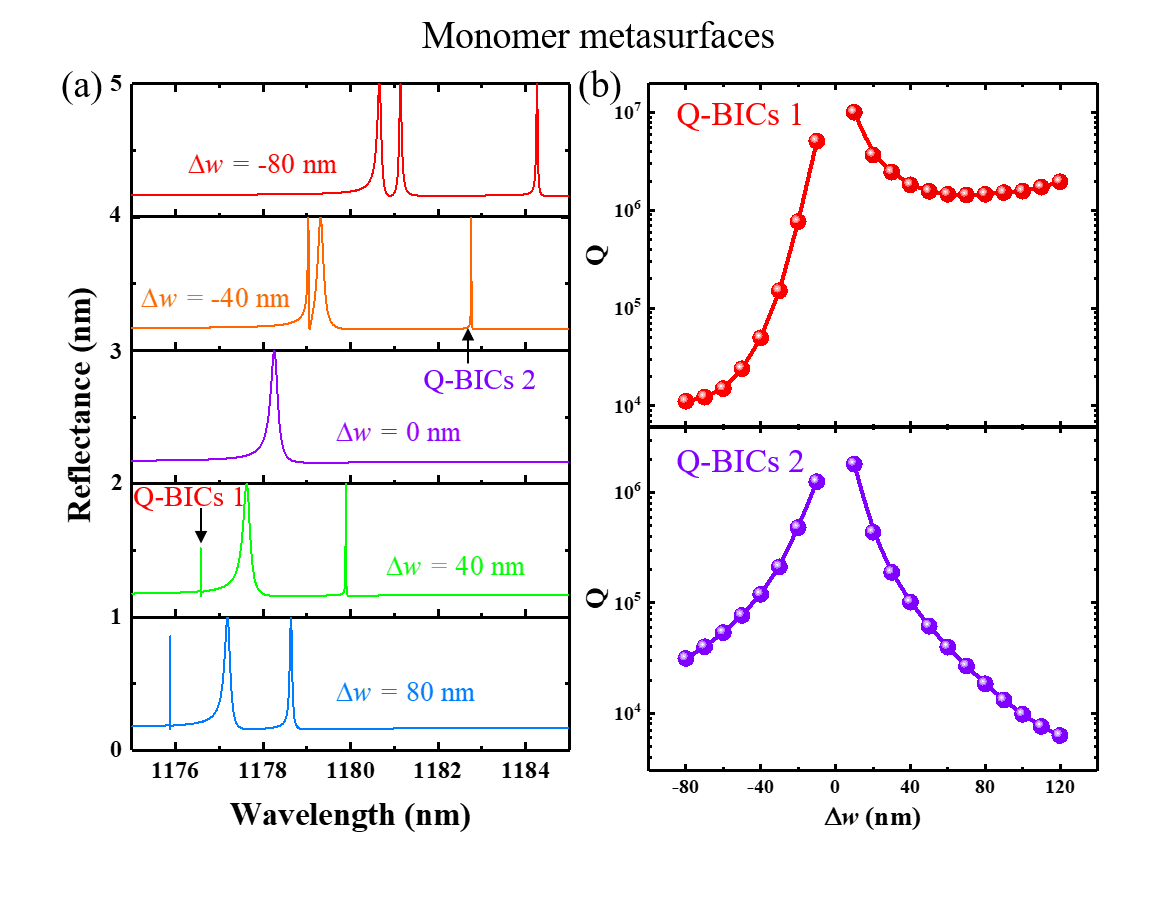


Figure S2. (a) Reflection spectra of monomer metasurfaces with different Δ*w*. (b) The extracted Q-factors as a function of Δ*w* for monomer metasurfaces. Here, *t*_wg_ = 140 nm, *t*_g_ = 60 nm, *w*_1_ = 280 nm, and Δ*w* = *w*_1_ − *w*_2_.


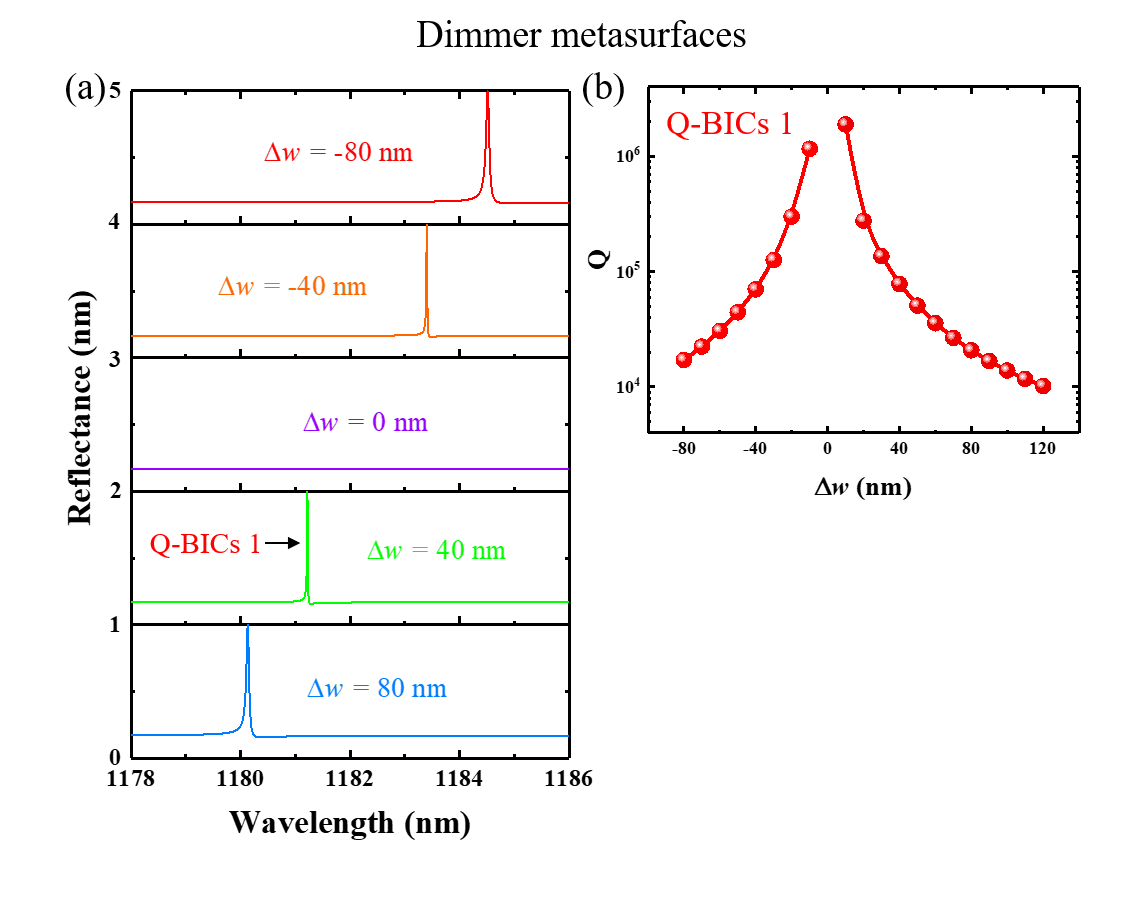


Figure S3. (a) Reflection spectra of dimmer metasurfaces with different Δ*w*. (b) The extracted Q-factors as a function of Δ*w* for dimmer metasurfaces. Here, *t*_wg_ = 140 nm, *t*_g_ = 60 nm, *w*_1_ = 280 nm, and Δ*w* = *w*_1_ − *w*_2_.


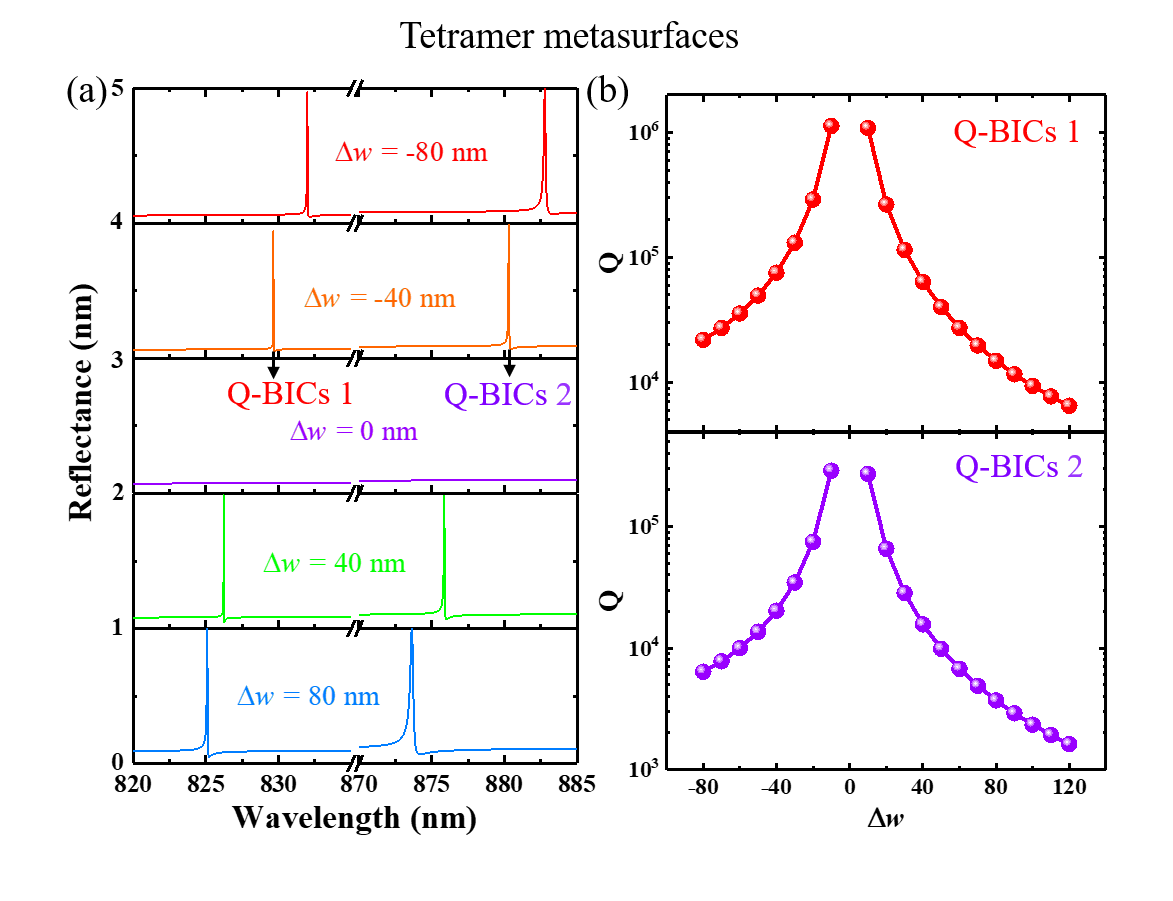


Figure S4. (a) Reflection spectra of tetramer metasurfaces with different Δ*w*. (b) The extracted Q-factors as a function of Δ*w* for tetramer metasurfaces. Here, *t*_wg_ = 140 nm, *t*_g_ = 60 nm, *w*_1_ = 280 nm, and Δ*w* = *w*_1_ − *w*_2_.

**Section S3. Surface sensitivity optimization.**


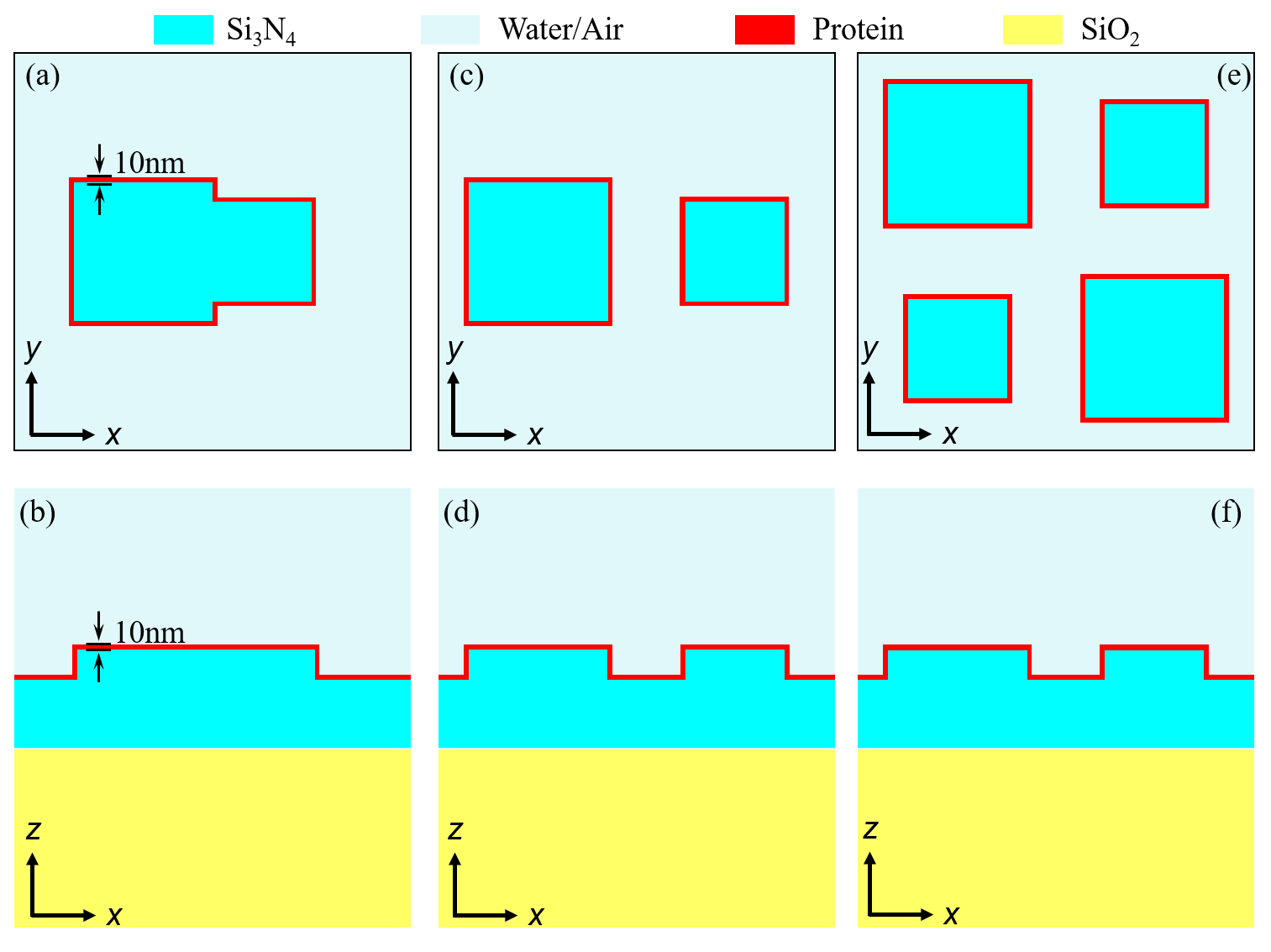


Figure S5. Schematic illustrations of the top-section and cross-section for (a, b) AMM, (c, d) ADM,

and (e, f) ATM structures with a protein layer with a thickness of 10 nm.

To further improve the surface sensitivity (*S*_surface_) of the designed metasurfaces, we optimized the structural parameters to seek for higher *S*_surface_. In the main text, a shallow *t*_g_ was used to compare the sensing performance for different meta-atom designs. Here, we investigated the evolution of *S*_surface_ under different *t*_g_ and their results were plotted in Figure S6. It was observed that *S*_surface_ could be improved by increasing *t*_g_ for all meta-atom designs. Importantly, the TM (1,1) mode of ATM structure exhibited a higher *S*_surface_ than other structures and the highest *S*_surface_ was 0.38 nm/nm at *t*_g_ = 300 nm.

Next, we attempted to investigate the influence of *t*_wg_ on *S*_surface_. Here, *t*_g_ was fixed at 300 nm and *t_w_*_g_ was varied from 60 nm to 160 nm. In Figure S7a, *S*_surface_ increased as *t*_wg_ decreased under TM (1,1) mode. For TE (1,1) mode, a maximum *S*_surface_ occurred at *t*_wg_ = 80 nm. However, it should note that a metasurface with thinner *t*_wg_ (<100 nm) cannot support TM (1,1) mode in the air environment (Figure. S7b), which is important for a variety of potential applications, such as nonlinear optical effects, optical switch, and so on. Hence, we selected the structural parameters of *t*_g_ = 300 nm and *t*_wg_ = 100 nm (called thick ATM) as our final optimized parameters. The *S*_surface_ of the thick ATM structure was ~0.4 nm/nm in the water environment, which was approximately 1.67 times higher than shallow ATM structure (*S*_surface_ = 0.239 nm/nm for shallow ATM).


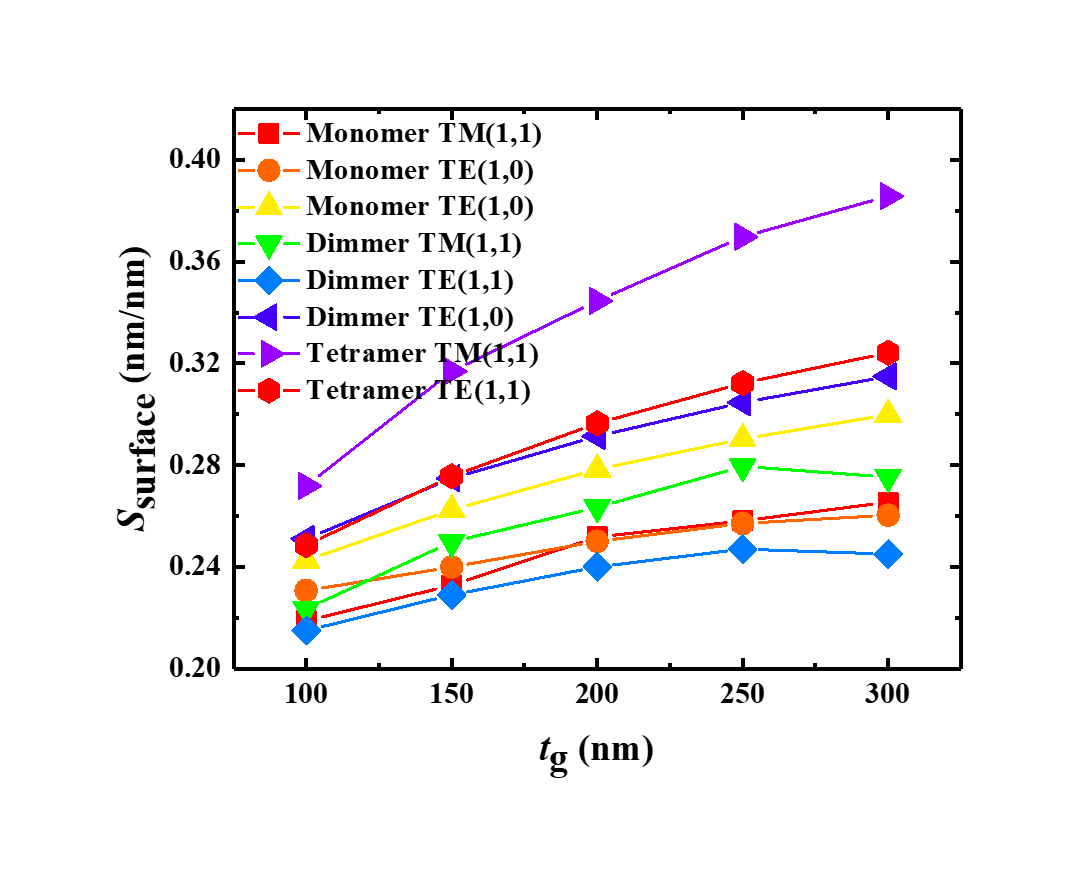


Figure S6. Calculated *S*_surface_ of different meta-atom designs as a function of *t*_g_. Here, *w*_1_ = 280 nm, *w*_2_ = 200 nm, and *t*_wg_ = 140 nm.


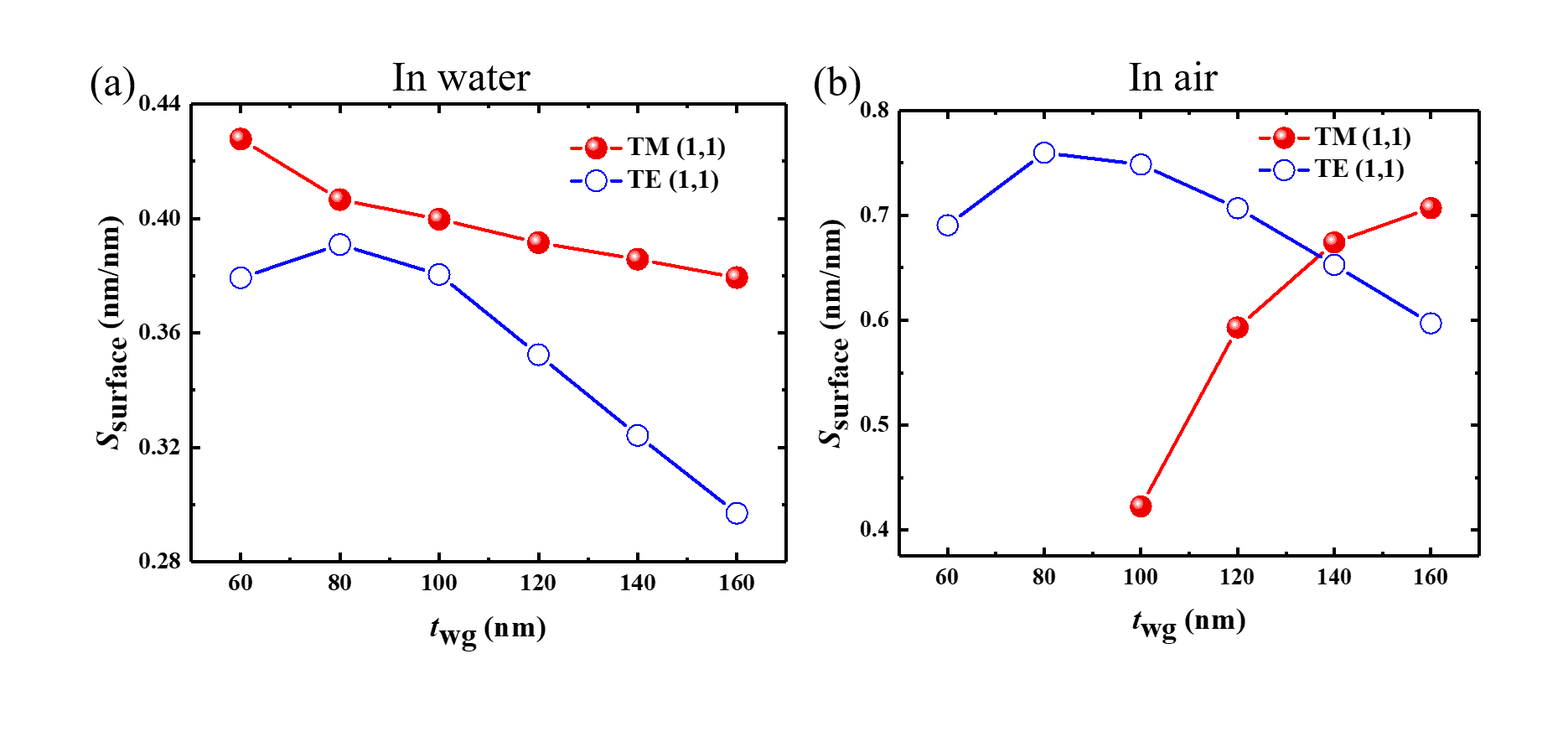


Figure S7. Calculated *S*_surface_ of ATM structure as a function of *t*_wg_ (a) in water and (b) in air. Here, *w*_1_ = 280 nm, *w*_2_ = 200 nm, and *t*_g_ = 300 nm.

**Section S4. Simulated reflection spectrum for ATM structures.**


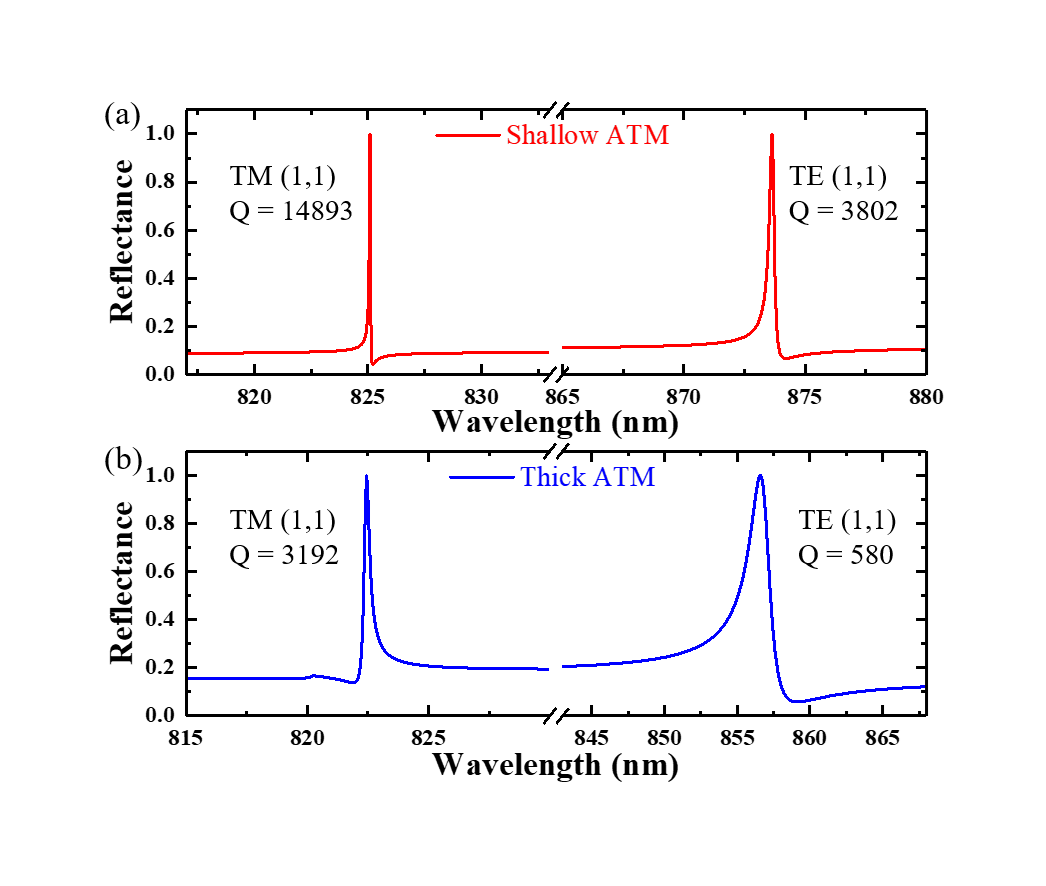


Figure S8. Simulated reflection spectrum for (a) shallow ATM and (b) thick ATM in air. For shallow ATM, *w*_1_ = 280 nm, *w*_2_ = 200 nm, *t*_g_ = 60 nm, and *t*_wg_ = 140 nm. For thick ATM, *w*_1_ = 280 nm, *w*_2_ = 200 nm, *t*_g_ = 300 nm, and *t*_wg_ = 100 nm.


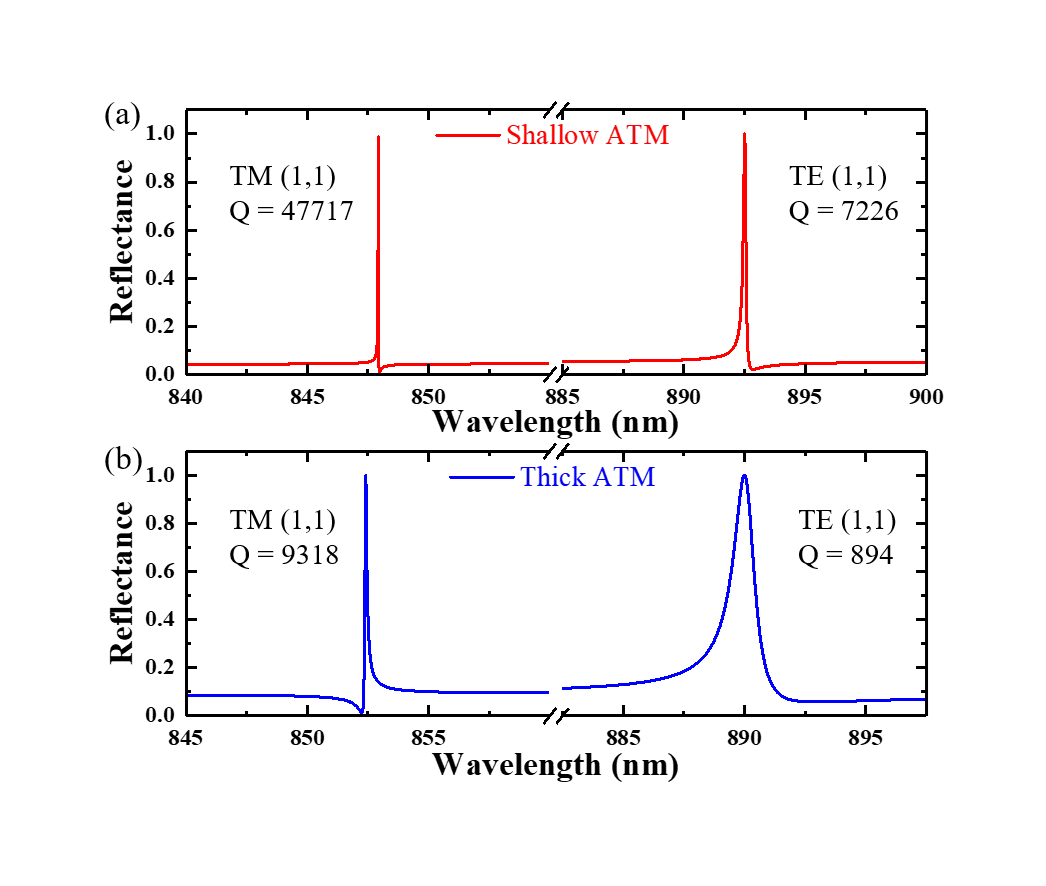


Figure S9. Simulated reflection spectrum for (a) shallow ATM and (b) thick ATM in water. For shallow ATM, *w*_1_ = 280 nm, *w*_2_ = 200 nm, *t*_g_ = 60 nm, and *t*_wg_ = 140 nm. For thick ATM, *w*_1_ = 280 nm, *w*_2_ = 200 nm, *t*_g_ = 300 nm, and *t*_wg_ = 100 nm.

**Section S5. Summary of measured thick ATM samples in air.**


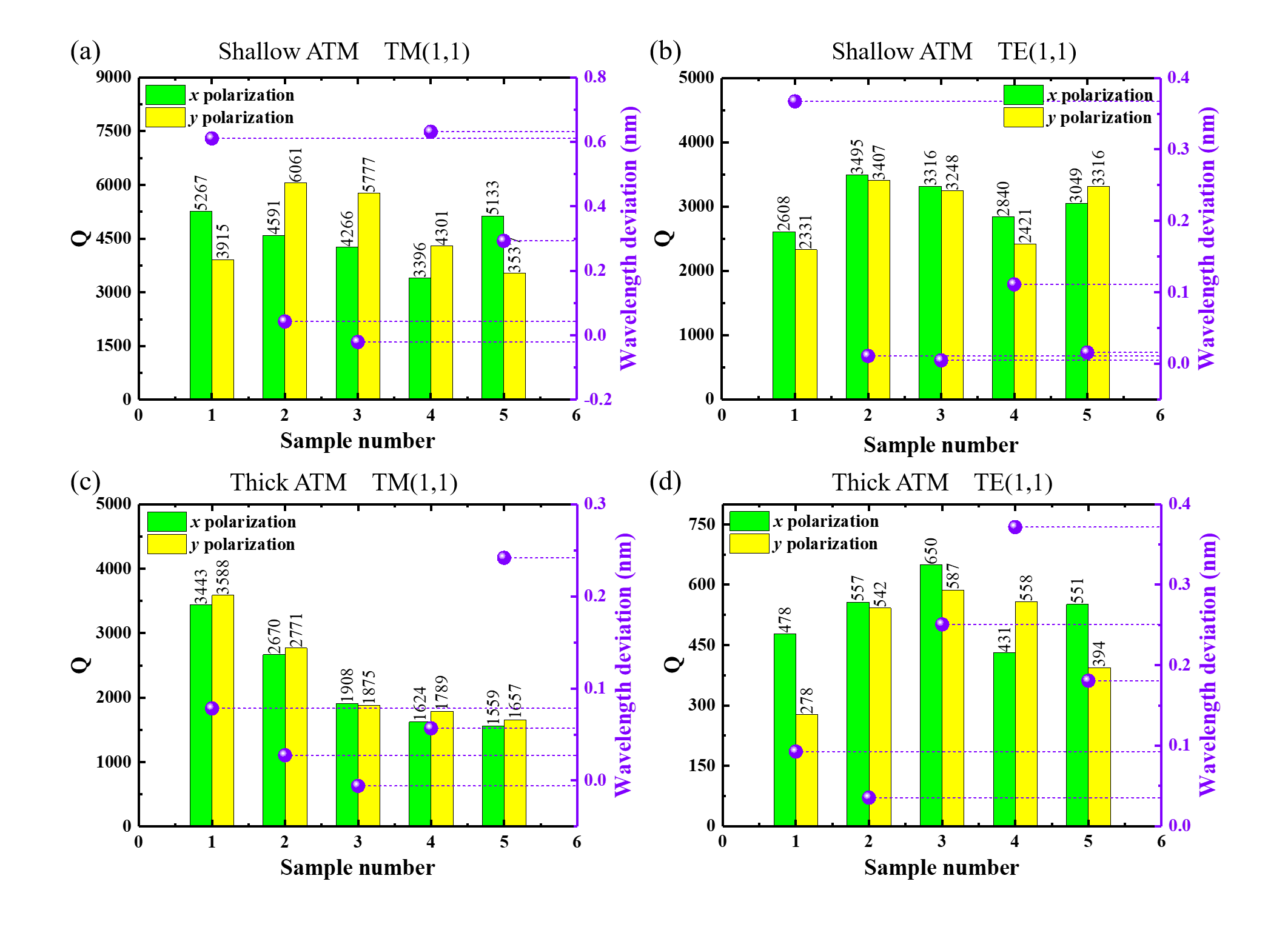


Figure S10. Statistics of measured Q-factors (Left Y-axis) and wavelength deviation (Right Y-axis) for the fabricated (a, b) shallow ATM and (c, d) thick ATM samples.

**Section S6. Effect of array size.**


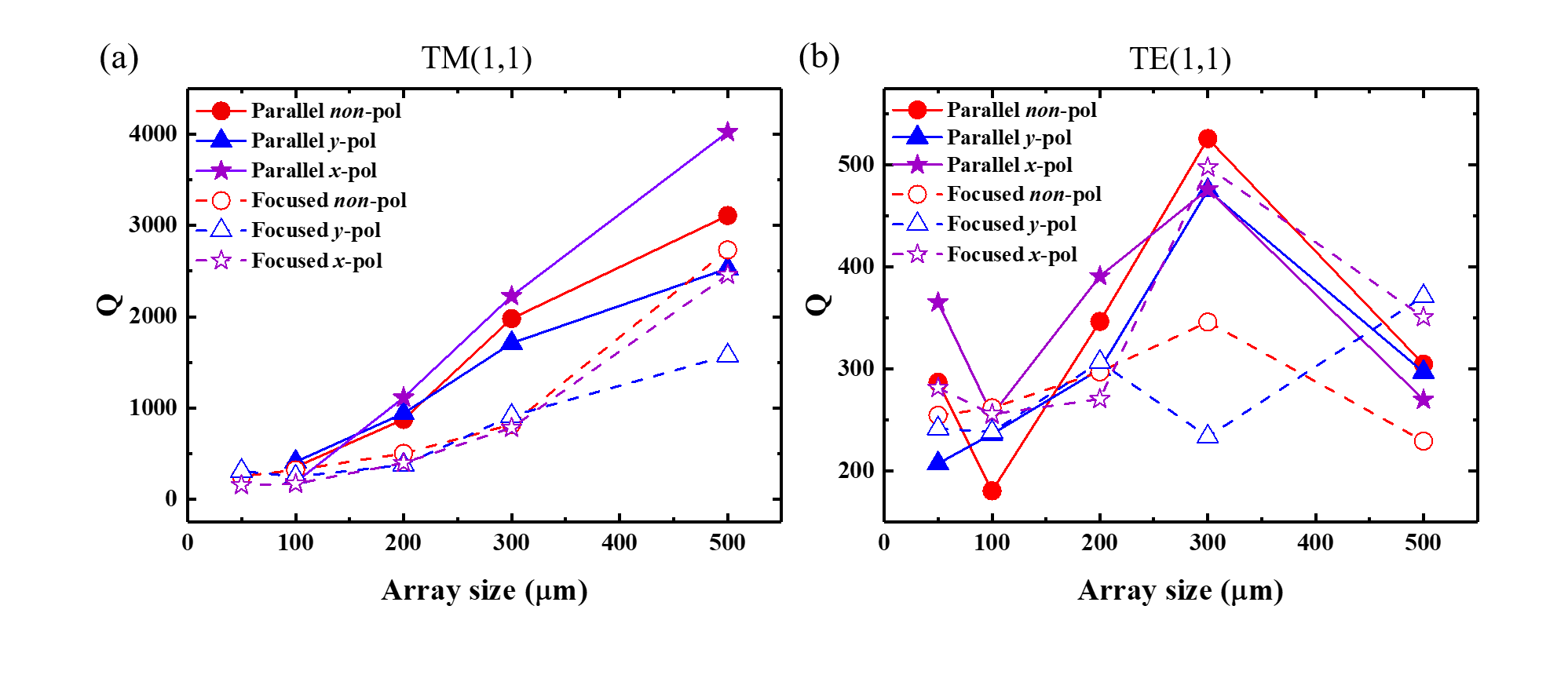


Figure S11. The extracted Q-factors of the thick ATMs as a function of array size.

**Table S1.** Summary of experimentally obtained optical properties in asymmetric metasurfaces governed by quasi-BICs.

| Types | Material | Defects size  (nm) | Wavelength  (nm) | *ζ* | *α* | Q-factor | References |
| --- | --- | --- | --- | --- | --- | --- | --- |
| Monomer | Si | 220 | 1345 | 0.16 | 0.29 | 128 | [1] |
| Monomer | Si | 60 | 760 | 0.08 | 0.34 | 120 | [2] |
| Monomer | Si | 160 | 1588 | 0.1 | 0.12 | 18511 | [3] |
| Monomer | Si | 140 | 1313 | 0.11 | 0.15 | 8911 | [4] |
| Monomer | Si | 180 | 1280 | 0.14 | 0.18 | \ | [5] |
| Dimer | Si | 100 | 850 | 0.12 | 0.3 | 144 | [6] |
| Dimer | Si | 100 | 835 | 0.12 | 0.44 | 250 | [7] |
| Dimer | Si | 195 | 1460 | 0.13 | 0.05 | 175 | [8] |
| Dimer | GaP | 200 | 1210 | 0.17 | 0.09 | 2000 | [9] |
| Trimer | Si | 115 | 1375 | 0.08 | 0.13 | 483 | [10] |
| Tetramer | TiO_2_ | 180 | 720 | 0.25 | 0.19 | ~1000 | [11] |
| Tetramer | **Si_3_N_4_** | **200** | **827** | **0.24** | **0.49** | **6061** | **This work** |

References

[1] L. Xu, K. Z. Kamali, L. J. Huang *et al.*, “Dynamic Nonlinear Image Tuning through Magnetic Dipole Quasi-BIC Ultrathin Resonators,”*Adv. Sci.,* vol. 6, p. 1802119, 2019.

[2] J. Wang, J. Kuhne, T. Karamanos *et al.*, “All-Dielectric Crescent Metasurface Sensor Driven by Bound States in the Continuum,” *Adv. Funct. Mater.,* vol. 31, p. 2104652, 2021.

[3] Z. J. Liu, Y. Xu, Y. Lin *et al.*, “High-Q Quasibound States in the Continuum for Nonlinear Metasurfaces,” *Phys. Rev. Lett.,* vol. 123, p. 253901, 2019.

[4] Z. J. Liu, J. Y. Wang, B. Chen *et al.*, “Giant Enhancement of Continuous Wave Second Harmonic Generation from Few-Layer GaSe Coupled to High-Q Quasi Bound States in the Continuum,” *Nano Lett.,* vol. 21, pp. 7405-7410, 2021.

[5] L. Q. Zhu, S. Yuan, C. Zeng, and J. S. Xia, “Manipulating Photoluminescence of Carbon G-center in Silicon Metasurface with Optical Bound States in the Continuum,” *Adv. Opt. Mater.,* vol. 8, p. 1901830, 2020.

[6] F. Yesilkoy, E. R. Arvelo, Y. Jahani *et al.*, “Ultrasensitive hyperspectral imaging and biodetection enabled by dielectric metasurfaces,” *Nat. Photonics,* vol. 13, pp. 390-396, 2019.

[7] Y. Jahani, E. R. Arvelo, F. Yesilkoy *et al.*, “Imaging-based spectrometer-less optofluidic biosensors based on dielectric metasurfaces for detecting extracellular vesicles,” *Nat. Commun.,* vol. 12, p. 3246, 2021.

[8] K. Koshelev, Y. T. Tang, K. F. Li *et al.*, “Nonlinear Metasurfaces Governed by Bound States in the Continuum,” *ACS Photonics,* vol. 6, pp. 1639-1644, 2019.

[9] A. P. Anthur, H. Z. Zhang, R. Paniagua-Dominguez *et al.*, “Continuous Wave Second Harmonic Generation Enabled by Quasi- Bound-States in the Continuum on Gallium Phosphide Metasurfaces,” *Nano Lett.,* vol. 20, pp. 8745-8751, 2020.

[10] Y. M. Yang, I. I. Kravchenko, D. P. Briggs, and J. Valentine, “All-dielectric metasurface analogue of electromagnetically induced transparency,” *Nat. Commun.,* vol. 5, p. 5753, 2014.

[11] P. Vaity, H. Gupta, A. Kala *et al.*, “Polarization‐Independent Quasibound States in the Continuum,” *Adv. Photonics Res.,* vol. 3, p. 2100144, 2021.
